# Investigating Voluntary Medical Male Circumcision Program Efficiency Gains through Subpopulation Prioritization: Insights from Application to Zambia

**DOI:** 10.1371/journal.pone.0145729

**Published:** 2015-12-30

**Authors:** Susanne F. Awad, Sema K. Sgaier, Bushimbwa C. Tambatamba, Yousra A. Mohamoud, Fiona K. Lau, Jason B. Reed, Emmanuel Njeuhmeli, Laith J. Abu-Raddad

**Affiliations:** 1 Infectious Disease Epidemiology Group, Weill Cornell Medical College in Qatar, Cornell University, Qatar Foundation, Education City, Doha, Qatar; 2 Integrated Delivery, Global Development Program, Bill & Melinda Gates Foundation, Seattle, Washington, United States of America; 3 Department of Global Health, University of Washington, Seattle, Washington, United States of America; 4 Ministry of Community Development and Mother and Child Health, Lusaka, Zambia; 5 Office of the U.S. Global AIDS Coordinator, Washington, District of Columbia, United States of America; 6 United States Agency for International Development, Washington, District of Columbia, United States of America; 7 Department of Healthcare Policy and Research, Weill Cornell Medical College, Cornell University, New York, New York, United States of America; 8 College of Public Health, Hamad bin Khalifa University, Qatar Foundation, Education City, Doha, Qatar; International AIDS Vaccine Initiative, UNITED STATES

## Abstract

**Background:**

Countries in sub-Saharan Africa are scaling-up voluntary male medical circumcision (VMMC) as an HIV intervention. Emerging challenges in these programs call for increased focus on program efficiency (optimizing program impact while minimizing cost). A novel analytic approach was developed to determine how subpopulation prioritization can increase program efficiency using an illustrative application for Zambia.

**Methods and Findings:**

A population-level mathematical model was constructed describing the heterosexual HIV epidemic and impact of VMMC programs (age-structured mathematical (ASM) model). The model stratified the population according to sex, circumcision status, age group, sexual-risk behavior, HIV status, and stage of infection. A three-level conceptual framework was also developed to determine maximum epidemic impact and program efficiency through subpopulation prioritization, based on age, geography, and risk profile. In the baseline scenario, achieving 80% VMMC coverage by 2017 among males 15–49 year old, 12 VMMCs were needed per HIV infection averted (effectiveness). The cost per infection averted (cost-effectiveness) was USD $1,089 and 306,000 infections were averted. Through age-group prioritization, effectiveness ranged from 11 (20–24 age-group) to 36 (45–49 age-group); cost-effectiveness ranged from $888 (20–24 age-group) to $3,300 (45–49 age-group). Circumcising 10–14, 15–19, or 20–24 year old achieved the largest incidence rate reduction; prioritizing 15–24, 15–29, or 15–34 year old achieved the greatest program efficiency. Through geographic prioritization, effectiveness ranged from 9–12. Prioritizing Lusaka achieved the highest effectiveness. Through risk-group prioritization, prioritizing the highest risk group achieved the highest effectiveness, with only one VMMC needed per infection averted; the lowest risk group required 80 times more VMMCs.

**Conclusion:**

Epidemic impact and efficiency of VMMC programs can be improved by prioritizing young males (sexually active or just before sexual debut), geographic areas with higher HIV prevalence than the national, and high sexual-risk groups.

## Introduction

Three randomized controlled trials in 2005 and 2007 demonstrated that circumcision reduces the risk of males acquiring HIV through female-to-male transmission by approximately 60% [1–3]. In settings with high HIV prevalence and low circumcision rates, voluntary medical male circumcision (VMMC) is an effective, cost-effective, and cost-saving HIV prevention intervention [4]. The World Health Organization (WHO) and the Joint United Nations Programme on HIV/AIDS (UNAIDS) recommend making VMMC part of a comprehensive HIV prevention package in countries with generalized HIV epidemics and low male circumcision rates [5]. With support from the international community and local governments, scale-up of VMMC for HIV prevention has been initiated in 14 countries in East and Southern Africa.

Different mathematical modeling studies have highlighted the potential gains of VMMC as an HIV intervention [4, 6–11]. Modeling using the Decision-Makers’ Program Planning Tool (DMPPT) has outlined the potential impact that could be attained from the scale-up of VMMC [4]: A scenario where 80% coverage among males 15–49 year old in 13 priority African countries is achieved within five years, and maintaining the coverage thereafter, was estimated to avert 3.4 million HIV infections through 2025, to require $2 billion U.S. dollars (USD; all subsequent references to currency are in U.S. dollars), and to result in net savings of $16.5 billion. In light of these results and to further accelerate VMMC scale-up, WHO/UNAIDS launched a Joint Strategic Action Framework calling for 80% circumcision coverage (20.2 million males) among males 15–49 years of age in 14 countries in East and Southern Africa by 2016 [12]. With the exception of a few, most countries have used similar parameters for their national targets.

Sgaier et al. recently summarized the achievements of the global VMMC program to date and outlined key programmatic challenges the program is facing [13]. While the global program has nearly tripled its output in recent years, from 3.2 million by the end of 2012 to approximately 9 million by the end of 2014, national cumulative targets for the end of 2016 (and their projected impact) are unlikely to be achieved by the end of the scale-up period. Achievement of national targets and the global target of 20.2 million circumcisions will require acceleration of efforts, an increase in funding, and a focus on greater program efficiency [13]. Program efficiency is defined in this context as optimizing the impact (Gain) of the VMMC program while minimizing its cost (Pain).

To explore opportunities for the VMMC programs to achieve greater efficiency, we evaluated the prioritization of subpopulations of males—optimizing targets so that the program focuses on the most impactful circumcisions to achieve the ultimate goal of averted HIV infections—as a key strategy for more efficient and effective scale-up of the VMMC program. This is a feasible concept, given that not all circumcisions contribute equally to public health outcomes [4]. The epidemic impact of circumcisions varies by a number of factors that are related to HIV incidence [4].

**Fig 1:**
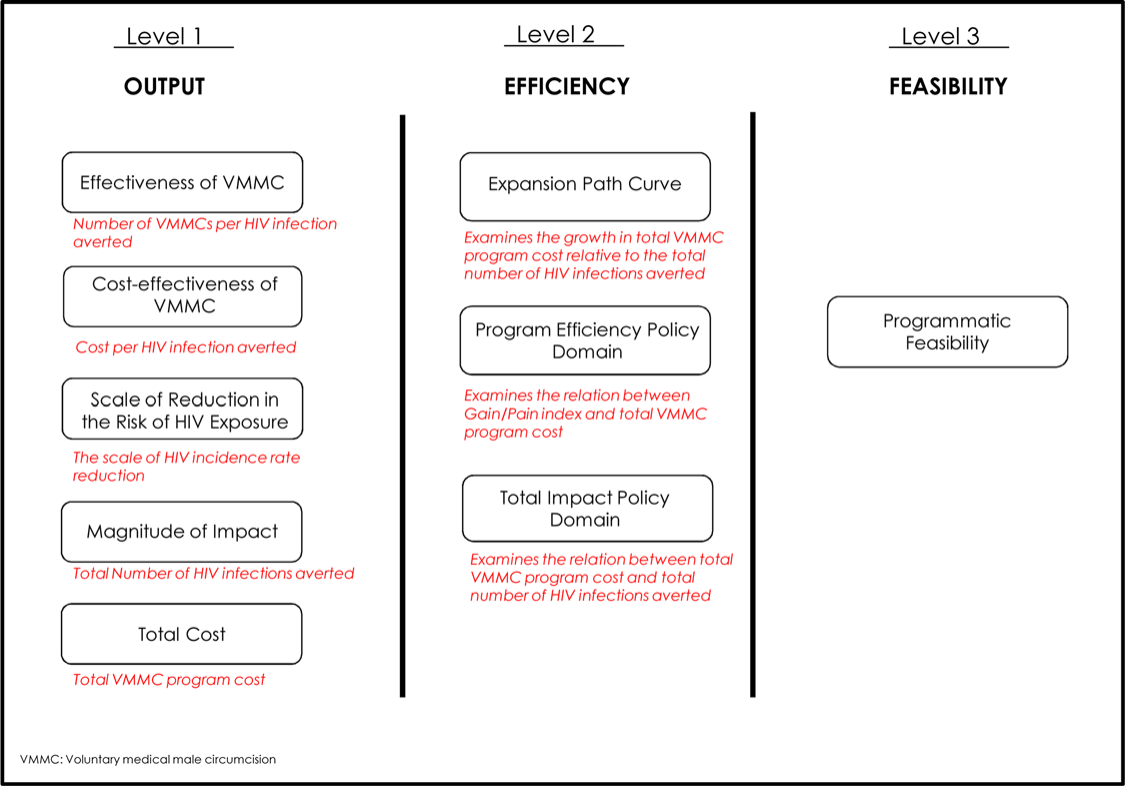
The three-level conceptual framework. The voluntary medical male circumcision (VMMC) program scenarios are assessed based on epidemiologic and health economics measures (Level 1), program efficiency and policy outcome measures (Level 2), and program feasibility (Level 3).

We developed the age-structured mathematical (ASM) model, a novel analytic framework, to describe the heterosexual HIV epidemic and to assess the impact of VMMC programs in sub-Saharan Africa (SSA). A novel three-level conceptual framework, complementing the ASM model, was also developed to ascertain whether it is possible to prioritize subpopulations based on age, geography, and risk profile of males to maximize programmatic efficiency (Fig 1). The first level of the framework assesses the VMMC program using epidemiologic and health economic measures (epidemic impact): 1) number of VMMCs needed to avert one HIV infection (*effectiveness*), 2) HIV incidence rate reduction, 3) absolute number of HIV infections averted (*magnitude of impact*), 4) cost per HIV infection averted (*cost-effectiveness*), and 5) total program cost. The second level of the framework assesses measures of program efficiency and policy outcomes using program expansion pathway curves [14] and policy-frontier plots. The frontier plots generated in this study provide, to our knowledge, also a novel method of visualizing and delineating seamless policy decision making. The third level of the framework is related to program feasibility. In this article, we present the methodological framework and results of its application to Zambia from a health system’s and donor’s perspectives.

## Methods

### Mathematical Model

We constructed a deterministic compartmental mathematical model, based on extension of earlier models [10, 15–19], that describes the heterosexual transmission of HIV in a given population—in this case the population of Zambia (S1 Text). Given the large scale HIV epidemic in Zambia, a deterministic model is an appropriate choice to examine HIV dynamics and intervention impact at the national level. The model consists of a set of coupled nonlinear differential equations that stratifies the population into compartments according to sex, circumcision status, age group, sexual risk group, HIV status, and stage of infection. HIV progression in the model is divided into the three stages of acute, chronic, and advanced infection.

The model disaggregates Zambia’s population into 20 age groups, with each group representing a five-year age band (0–4, 5–9, …, 95–99 year old). In order to account for heterogeneity in sexual risk behavior, we incorporated six sexual risk groups in the population, starting from lower to higher levels of sexual risk behavior (Risk group 1 being the lowest and Risk group 6 being the highest). It is assumed that people stay in the same risk group throughout their sexual activity lifespan (15–49 years of age). The distribution of the population across the risk groups follows a gamma distribution based on empirical data of the distribution of the number of sexual partners from SSA [20–23]. The level of sexual-risk behavior for each risk group is defined by the effective partnership change rate, which is a measure of the risk of exposure to HIV infection [16]. The distribution of the level of sexual-risk behavior across the different risk groups follows a power-law function based on a combination of theoretical analyses applied to sexual networks and empirical evidence [24–29].

We describe partner choice or mixing between the sexual risk groups and age groups by mixing matrices that incorporate both an assortative component (where partners are chosen preferentially from within the same risk or age group, for instance, low risk with low risk and high risk with high risk), and a proportionate component (where partners are chosen with no preferential bias based on risk or age group). For age mixing, we assumed males from a specific five-year age group will preferentially mix with females in the five-year age group below their age group (younger females), based on empirical data [30, 31].

A fraction of males in the population are assumed to be circumcised (baseline circumcision) through background traditional circumcision. The efficacy of VMMC against HIV acquisition is modeled as a proportional reduction in the risk of HIV acquisition among circumcised males. The model accommodates risk compensation that may theoretically be experienced by circumcised males following VMMC, through a proportional increase in the level of sexual risk behavior. Nonetheless, given the inconclusive empirical evidence to support increases in risk behavior following VMMC [32–36], we assumed 0% risk compensation in our main predictions. The modeling analyses were conducted using MATLAB version R20015a [37]. Further details on the model structure and assumptions can be found in S1 Text.

### Data Sources

The model was parameterized using empirical epidemiological and natural history data from SSA. The model’s parameter values along with their references are listed in S1 Table. The country-specific time series of HIV prevalence data was obtained from UNAIDS estimates that use a distillation of large and diverse sources of HIV prevalence data to arrive at a national-level trajectory for HIV prevalence [38]. Baseline male circumcision (non-VMMC program circumcisions) and HIV prevalence for each province in Zambia were obtained from the *Zambia Demographic and Health Survey (DHS) 2007*, which is a nationally representative household-based survey [39, 40]. Further data on HIV prevalence by province was obtained from Zambia’s 2011 HIV sentinel surveillance-antenatal clinics (HSS-ANC) data [41]. Demographics such as total population size and its future projections were obtained from the database of the Population Division of the United Nations Department of Economic and Social Affairs [42]. The VMMC unit cost per age group used in the model is shown in S2 Table. Two VMMC unit costs were assumed: a fixed VMMC unit cost of $95 across all age groups, and a breakdown of VMMC unit cost per age group. The latter cost structure was based on VMMC program data from Zambia [43]. Following convention, we applied an annual discount rate of 3% on future expenditures [44].

### Model Fitting

The model was fitted to HIV prevalence time series data using a nonlinear, least-square fitting method. This technique minimizes the sum of squares between all data points and the model, using the Nelder-Mead simplex algorithm as described in Lagarias *et al*. [45]. There has been substantial changes in the risk of HIV exposure across SSA and large reductions in HIV incidence [16]. To fit the model to HIV prevalence trend data in Zambia, the overall level of sexual risk behavior was assumed to vary during the HIV epidemic. Further details on the sexual risk behavior parametrization and its time evolution can be found in S1 Text.

### Conceptual Framework: Epidemiologic, Health Economics, Program Efficiency, and Policy Measures

The implications of the VMMC program were assessed through epidemiologic, health economics, program efficiency, and policy measures (the conceptual framework, Fig 1). The epidemiological metric used for the *effectiveness* of the VMMC intervention was the number of VMMCs required to avert one HIV infection. This measure was calculated by dividing the number of VMMCs by the number of infections averted over the chosen time horizon.

The metric used for the *scale of reduction in the risk of HIV exposure* is HIV incidence rate reduction. This measure was calculated by comparing the population-level HIV incidence rate in the presence of the intervention with a counter-factual scenario with no intervention.

The metric used for the *magnitude of the impact* of the VMMC intervention was the total number of HIV infections averted over the chosen time horizon. This measure was calculated by comparing the number of HIV infections in the presence of the intervention with a counter-factual scenario with no intervention. These epidemiologic measures (as part of the first level of the assessment framework), among others, were assessed at different points within the chosen time horizon. We applied an annual discount rate of 3% on future savings (HIV infections averted) [46].

The health economics metric used for the *cost-effectiveness* of the VMMC intervention was the cost to avert one HIV infection. This measure was calculated by dividing the total cost of the VMMC program by the total number of HIV infections averted over the chosen time horizon. Implicitly therefore the cost-effectiveness is the incremental cost-effectiveness ratio relative to a scenario of no VMMC intervention. The metric used for the *total program cost* was the actual VMMC program cost of delivering these VMMCs, including all direct and indirect costs related to the provision of VMMC such as personnel, VMMC commodities, and training. All costs were calculated in USD.

Two metrics were used to determine the *program efficiency* of different VMMC programs (the second level of the assessment framework). The first metric of program efficiency was the VMMC *public health program expansion path curve* [14]. This metric describes the growth in the total cost of the program as a function of the total number of HIV infections averted for the different subpopulation prioritization schemes. The hierarchy of subpopulation expansion was based on VMMC effectiveness; that is, the expansion path curve was generated by first targeting the subpopulation with the highest effectiveness, then adding the subpopulation with the second highest effectiveness, and so forth, up to the population with the least effectiveness. Accordingly, this metric provides a pathway for how a VMMC program can be efficiently expanded as feasible by total VMMC program cost. The second program efficiency metric was what we call the “Gain/Pain index”, defined as the proportional reduction in the total number of infections averted (Gain) over the proportional reduction in the total cost of the VMMC program (Pain). These proportions were assessed relative to the baseline (reference) scenario of targeting males aged 15–49 years.

The above measures were used collectively to generate *policy domains* for decision making using *policy-frontier plots*. Two policy themes were used to generate these frontier plots. The first frontier plot was based on the theme of maximizing program efficiency (maximizing gain while minimizing cost), thereby displaying the Gain/Pain index versus the total VMMC program cost. Within the plot, the different subpopulation prioritizations were classified into four distinct policy domains, based on being favorable or unfavorable in terms of Gain/Pain and total VMMC program cost.

The second policy-frontier plot was based on the theme of maximizing the total impact of the VMMC program, thereby displaying the total number of HIV infections averted versus the total VMMC program cost, but also factoring in cost-effectiveness. Within the plot, the different subpopulation prioritizations were classified into three distinct policy domains, based on being favorable or unfavorable in terms of total VMMC program cost, total impact, and cost-effectiveness.

The third level of the conceptual framework is programmatic feasibility, and is related to on-the-ground country experiences and national health policy priorities.

### Intervention Program Scenarios

#### Feasibility of the original Zambia VMMC scale-up plan

The original implementation strategy of the Zambia VMMC program scale-up aimed to reach 1.95 million VMMCs in a catch-up phase (2010–2015), by targeting the entire 15–49 year old uncircumcised male population [47]. The program’s scale-up plan included 84,604 VMMCs in 2011 and up to nearly 870,000 VMMCs in 2015 (S3 Table) [47]. We investigated the feasibility of achieving this scale-up plan target of 1.95 VMMCs among the 15–49 year age group through two forecast scenarios with different assumptions of the scale-up pace.

- Forecast plan based on no-growth scale-up scenario: We used the actual number of VMMCs that the VMMC program achieved between 2007 and 2013 [48], and then assumed that the number of VMMCs would be maintained per year at the 2013 level (294,466 VMMCs) up to the end of the catch-up phase in 2015.
- Forecast plan based on current VMMC program scale-up: We used the actual number of VMMCs that the program achieved between 2007 and 2013 [48], and supplemented these with the expected number of VMMCs for 2014 and 2015 as anticipated by the actual program [49]. In this scenario, the expected numbers of VMMCs year by year after 2013 are not dependent on the previous years, in contrast with the no-growth scenario, but are dependent on programmatic feasibility.

S3 Table summarizes the total number of estimated VMMCs, year by year, for each of the two scenarios compared with the original Zambia VMMC scale-up plan.

#### Baseline VMMC intervention scenario

We modeled the HIV epidemic in Zambia and the scale-up of a VMMC program initiated in 2010. The uptake of VMMC among HIV-negative and HIV-positive males was based on program data for Zambia. Consequently, less than 1% of those circumcised in the modeled scenarios were HIV-positive [48].

The intervention starts with a “catch-up” phase that lasts seven years rather than five (ending in 2017 instead of 2015 as in Zambia’s original implementation strategy). In this modeled baseline intervention scenario (reference scenario), the catch-up phase focuses on the 15–49 year old and ends by reaching 80% VMMC coverage in this population. This is consistent with the Government of Zambia’s original VMMC scale-up strategy following the WHO and UNAIDS recommendation [5, 12]. This scenario has been adopted as the baseline reference scenario in the ongoing efforts for assessing gains in VMMC program efficiency through subpopulation prioritization in SSA [13, 50].

The scale-up of VMMC during the catch-up phase was assumed to be at a fixed rate—that is, the likelihood of any male within the targeted subpopulation to be circumcised is fixed throughout the catch-up phase.

At the end of the catch-up phase, a new “sustainability” phase begins, lasting up to 2025 in some projections and up to 2045 in other projections, where the attained coverage achieved during the catch-up phase is maintained by circumcising the incoming cohorts into the population of 15-year-olds (those turning 15 in each year). We based our predictions for the impact of subpopulation targeting with reference to this modified version of the original Zambia VMMC program scale-up.

The intervention’s impact on the epidemic was evaluated in three time horizons: a short-term time horizon up to the end of the catch-up phase (2010–2017), an intermediate-term time horizon nearly a decade into the sustainability phase (2010–2025), and a long-term time horizon nearly three decades into the sustainability phase (2010–2045). In our analyses we mainly focused on the intermediate term, as the goal of the VMMC program is to impact the HIV epidemic in the near future, aligned with UNAIDS 2030 global epidemic impact goals [51].

#### Subpopulation targeting

We focused on three subpopulation targeting scenarios based on age, geographic location, and sexual risk profile.

Age-group prioritization: In this targeting scenario, we prioritized the VMMC intervention to specific age groups instead of the entire population of 15–49 year old. For each age-group targeting scenario, we assumed 80% VMMC coverage achieved by the end of 2017. We investigated age-group targeting through two approaches. In the first approach, we targeted each five-year age band (10–14, 15–19, …, 45–49 year old). In the second approach, we targeted wider age brackets relevant to programming and policy considerations (such as 10–29, 15–24, and 15–29 year old, among others). Because programmatic experience indicates that 39% of VMMCs had been provided to males who were <15 year old, we also included an age band for males 10–14 year old, in the interest of comparing the relative advantages of scale-up across all adolescent and adult age ranges.

Geographic prioritization: In this targeting scenario, we prioritized the VMMC intervention to males based on the distribution of HIV infection across the provinces in Zambia. We examined this targeting in three subscenarios. In the first one, we selected four provinces with HIV prevalence higher than the national HIV prevalence (Central, Copperbelt, Lusaka, and Western; S1 and S2 Figs). In the second subscenario, we included the two provinces with the highest HIV prevalence (Central and Lusaka). In the third subscenario, we included only Lusaka—the province with the highest HIV prevalence. In each of these subscenarios, we assumed 80% VMMC coverage among the 15–49 year old males by the end of 2017 in the targeted provinces. The distribution of HIV prevalence at the provincial level was determined using the 2011 HSS-ANC data [41] and the 2007 DHS data (S1 Fig). More details on the selection of provinces can be found in S2 Text.

Risk-group prioritization: In this targeting scenario, we prioritized the VMMC intervention to males based on their sexual risk-behavior profile—that is, according to their specific risk group. For each risk-group targeting scenario, we assumed 80% VMMC coverage among the population of 15–49 year old in this risk group by 2017.

### Uncertainty Analyses

For each five-year age-group targeting scenario, a multivariate uncertainty analysis was conducted to specify the ranges of uncertainty in the effectiveness of VMMC with respect to variations in the structural parameters of the model. This was done using Monte Carlo sampling from uniform probability distributions for the uncertainty in the biological and behavioral parameters of the model, and assuming an uncertainty of 20% around the point estimates of all parameters (S1 Table). This uncertainty range in input parameters was informed by the range of available data in the literature [16, 52, 53]. Accordingly, we varied HIV transmission probability per coital act, duration of each HIV stage, frequency of coital acts per HIV stage, duration of partnership, degree of assortativeness, scale parameter in the gamma distribution of the population across the risk groups, and exponent parameter in the power-law function of the distribution of sexual-risk behavior. Each set of new parameters generated by the Monte Carlo sampling was used to refit the HIV prevalence trend for Zambia and then assess the effectiveness using the new fitted model. We implemented 500 uncertainty runs of these fitted models for each five-year age-group. Based on that we determined the geometric mean value and associated 95% uncertainty interval for the effectiveness of the VMMC intervention.

### Sensitivity Analyses

Future predictions of program impact can change depending on yet unknown factors such as the future scale-up of antiretroviral therapy (ART) and other interventions. Accordingly, we conducted two sensitivity analyses on our model predictions. In the first sensitivity analysis, we explored VMMC effectiveness in the presence of an optimistic roll-out of ART, as highlighted by recent WHO projections of reducing HIV incidence by 72% [54]. In the second sensitivity analysis, we assessed the sensitivity of our predictions to sexual-risk behavior compensation among those circumcised. Risk compensation ranging from 0% up to 25% was explored, and the predictions for VMMC effectiveness were compared to the core scenario assuming no risk compensation.

## Results

### Feasibility of the Original Zambia VMMC Scale-up Plan

**Fig 2:**
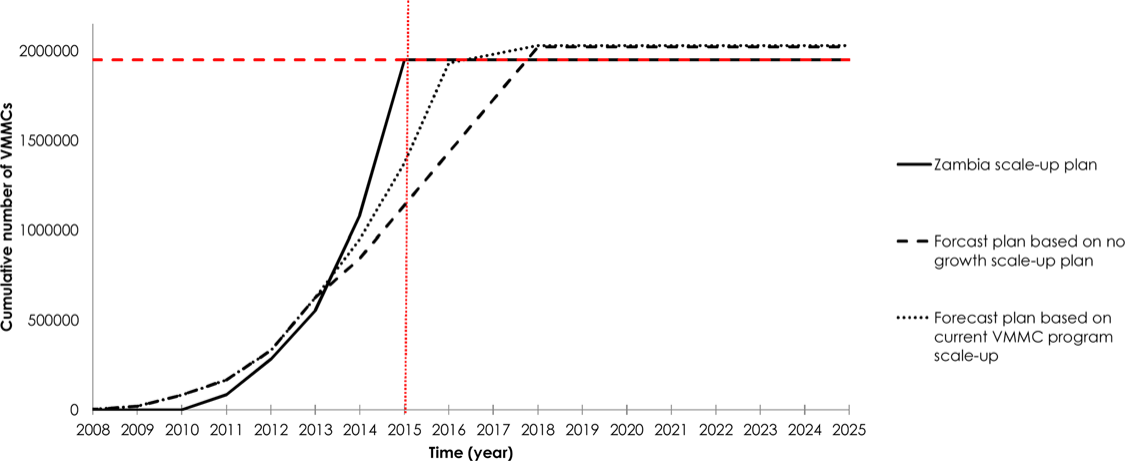
Feasibility and time required to achieve 1.95 million voluntary medical male circumcisions (VMMCs) among 15–49 year old males through two forecast scenarios. The cumulative number of VMMCs for two different projections based on the current roll-out of the VMMC program (*forecast plan based on no-growth scale-up* scenario and *forecast plan based on current VMMC program scale-up* scenario). These projections are compared to the original Zambia VMMC program scale-up plan.

Fig 2 and S3 Table explore the feasibility of achieving 1.95 million VMMCs among 15–49 year old males by 2015 through the two forecast scenarios. Each scenario was compared to the original Zambia VMMC program scale-up plan [47] (S3 Table). The forecast plan based on current actual VMMC program scale-up achieved 1.37 million VMMCs by 2015. The predicted number of VMMCs was lower in the forecast plan based on the no-growth scenario (1.21 million). Accordingly, by the year 2015, the two forecast scenarios would have achieved only 62% to 70% of the original Zambia VMMC scale-up plan’s target of 1.95 million VMMCs, and only 61% of implemented VMMCs would actually be among 15–49 year old males [4, 47]. In these forecast scenarios, the VMMC program would have achieved only 25% to 30% VMMC coverage among the 15–49 year old males by 2015. The two forecast scenarios would reach the goal of 1.95 million VMMCs only if the VMMC plans continue implementing circumcisions for two to three more years.

### Baseline Reference Scenario

Given predicted growth in population size (S3 Fig), a total of 2.53 million VMMCs would be required in the catch-up phase to achieve 80% VMMC coverage by 2017 among the 15–49 year old males (Table 1). Based on Zambia’s population pyramid, 580,000 additional VMMCs would be required to achieve 80% coverage by 2017, compared to the original goal of 1.95 million by 2015. An additional 1.21 million VMMCs would be required in the sustainability phase between 2018 and 2025 in order to maintain this coverage level. At this VMMC coverage level, 24% of new HIV infections would be averted between 2010 and 2025. This translates into approximately 306,000 HIV infections averted, with approximately 23 VMMCs needed to avert one infection in the short term (2010–2017) and 12 in the intermediate term (2010–2025). Given the variable cost structure per unit VMMC (S2 Table), the total cost of VMMC program scale-up by the year 2025 would be around $330 million.

**Table 1:** Epidemic impact of prioritizing different age groups in the voluntary medical male circumcision (VMMC) program.

Age group	#VMMC/HIA (2010–25)	#VMMCs (2010–17) (millions)	Additional VMMCs (2018–25) (millions)	HIA (millions) (2010–25)	Cost/HIA (2010–25)	Total cost (billion) (2010–25)
**15**–**49**	**12**	**2.53**	**1.2**	**0.31**	**1,089**	**0.33**
10–14	23	1.28 (51%)	1.4	0.11 (38%)	1,759 (161%)	0.20 (61%)
15–19	13	1.12 (44%)	1.2	0.18 (58%)	1,045 (96%)	0.18 (56%)
20–24	11	0.95 (38%)	1.0	0.19 (61%)	888 (81%)	0.16 (50%)
25–29	13	0.78 (31%)	0.9	0.13 (42%)	1,117 (103%)	0.14 (43%)
30–34	15	0.63 (25%)	0.7	0.09 (29%)	1,396 (128%)	0.12 (37%)
35–39	19	0.51 (20%)	0.6	0.06 (21%)	1,561 (143%)	0.10 (30%)
40–44	24	0.48 (19%)	0.4	0.04 (12%)	2,187 (201%)	0.08 (24%)
45–49	36	0.34 (13%)	0.4	0.02 (7%)	3,300 (303%)	0.07 (20%)
15–24	12	1.52 (60%)	1.3	0.24 (78%)	974 (89%)	0.23 (70%)
15–29	12	1.80 (71%)	1.3	0.27 (87%)	977 (90%)	0.26 (78%)
15–34	12	2.04 (81%)	1.3	0.29 (94%)	996 (97%)	0.28 (85%)
10–24	15	2.11 (84%)	1.6	0.24 (80%)	1,206 (111%)	0.29 (88%)
10–29	14	2.40 (95%)	1.6	0.28 (91%)	1,167 (107%)	0.32 (97%)
10–34	14	2.64 (104%)	1.5	0.30 (97%)	1,154 (106%)	0.34 (103%)
10–49	14	3.16 (125%)	1.3	0.33 (107%)	1,343 (123%)	0.44 (131%)

The number of VMMCs needed to avert one HIV infection (2010–2025) (*effectiveness*); the total number of VMMCs needed to reach 80% coverage by 2017; the additional number of VMMCs needed during the sustainability phase (2018–2025); the total number of HIV infections averted (2010–2025) (*magnitude of impact*); the cost needed to avert one HIV infection (2010–2025) (*cost-effectiveness*); and the total program cost (2010–2025) (*program cost*). The 15–49 year old male population is used as the baseline VMMC intervention scenario for comparison purposes. The numbers in parentheses indicate the fractions achieved relative to the baseline.

VMMC: Voluntary medical male circumcision, HIA: HIV infection(s) averted.

### Age-Group Prioritization

**Fig 3:**
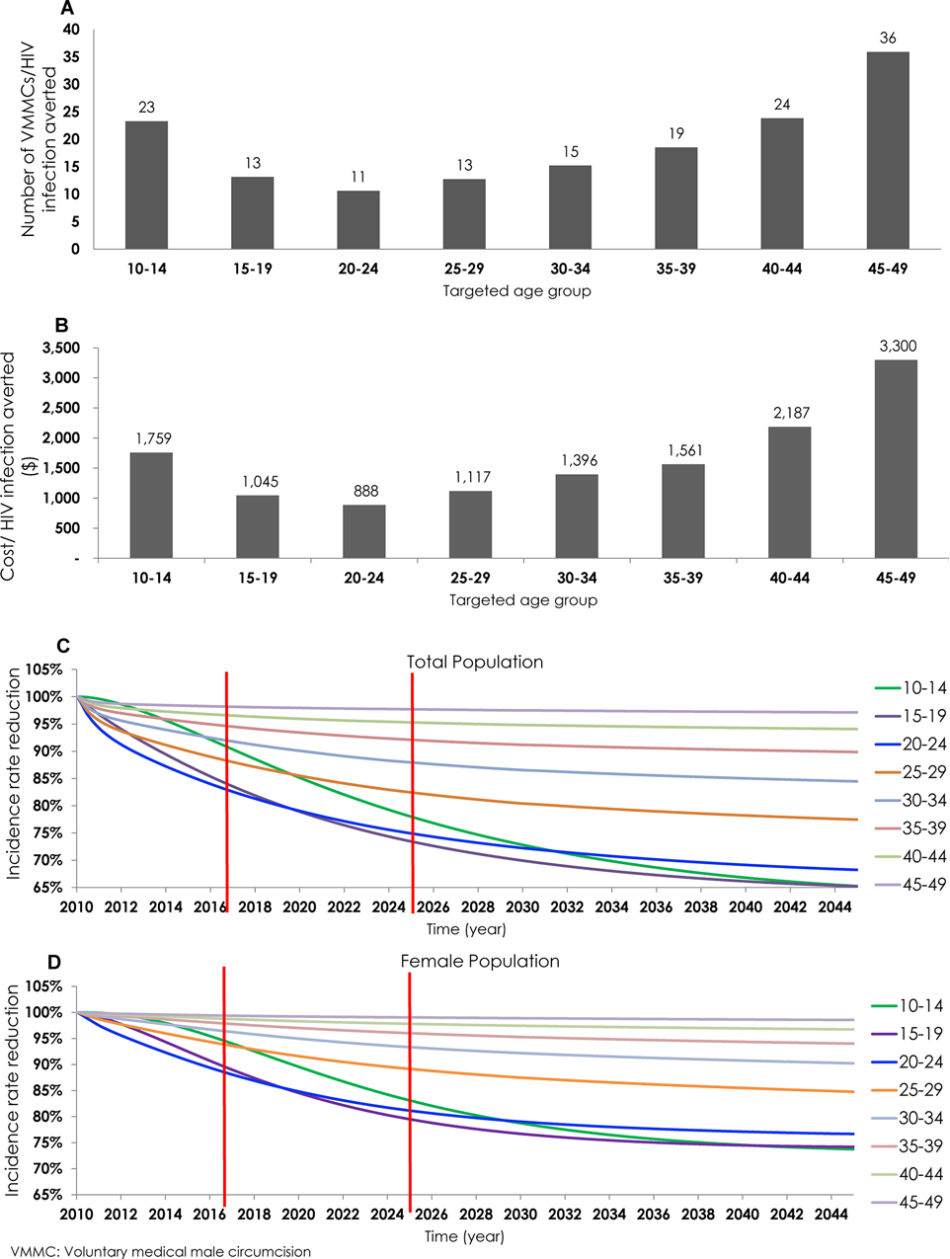
Projected outcomes of age-group prioritization. **A)** Number of voluntary medical male circumcisions (VMMCs) needed to avert one HIV infection (*effectiveness*) by 2025. **B)** Cost per HIV infection averted by 2025 (*cost-effectiveness*). **C)** Proportion of incidence rate reduction in the total adult population throughout the years up to 2045. **D)** Proportion of incidence rate reduction in the female adult population throughout the years up to 2045. The results are for 80% VMMC coverage by 2017 in the prioritized age group.

The age-stratified results of effectiveness of reaching 80% VMMC coverage are illustrated in Fig 3A. In the intermediate term (through 2025), by targeting each of the five-year age bands separately, the number of VMMCs needed to avert one HIV infection ranged from 11 to 36. The highest effectiveness was achieved by targeting the 20–24 year old age group, with 11 VMMCs needed per HIV infection averted. The second highest effectiveness was achieved by targeting the 15–19 or 25–29 year old age groups, with 13 VMMCs per infection averted. The VMMC program showed lower effectiveness by targeting males in the younger age group of 10–14 year old (23 VMMCs per infection averted), and age groups of those 30 years of age and older. The lowest effectiveness was by targeting males 45–49 year old (36 VMMCs per infection averted).

Fig 3B demonstrates that the cost-effectiveness of age-group targeting measured by program cost per infection averted followed the same pattern as the effectiveness of age group targeting measured by VMMCs per infections averted. The VMMC program cost per infection averted ranged from $888 (age group 20–24) to $3,300 (age group 45–49).

As part of the first level in the assessment framework (Fig 1), we also examined the incidence rate reduction through age-group targeting. Fig 3C shows the impact of age-group targeting on HIV incidence rate in the total adult population (15–49 year old). In the short term (by 2017), the incidence rate in the population was reduced by as much as 17%, with the largest reductions observed by targeting males in the 15–19 and/or 20–24 age groups. This changed in the intermediate term (by 2025), where the incidence rate was reduced by as much as 27%, with the largest reductions observed by targeting males in the 10–14, 15–19 and/or 20–24 age groups. In the long term, by 2045, the incidence rate was reduced by as much as 35%, with the largest reductions observed by targeting males in the 10–14, 15–19, and/or 20–24 age groups.

As was the case in the total adult population, in the short term the largest reductions in the incidence rate in the female adult population were achieved by targeting males 15–24 years of age (12%; Fig 3D), while in the intermediate term, the largest reductions were achieved by targeting males 10–24 years of age (20%). In the long term, the incidence rate in the female population was reduced by as much as 26%, with the largest reductions observed—as with the total adult population—by targeting males in the 10–14, 15–19, and/or 20–24 age groups.

**Fig 4:**
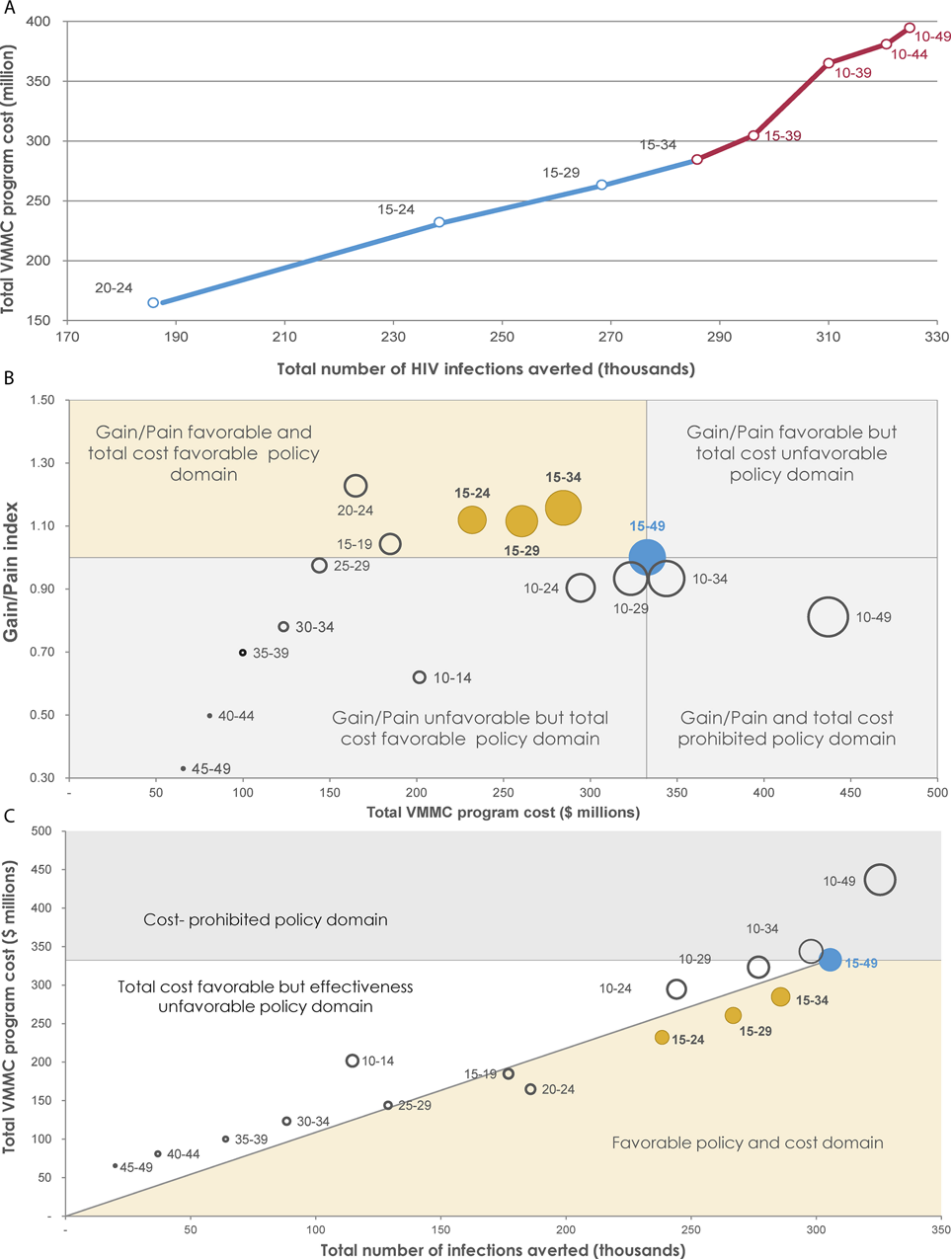
Program efficiency and policy domains of age-group prioritization in the voluntary medical male circumcision (VMMC) program. **A)** Expansion path curve showing the incremental increase in total cost of the VMMC program relative to total number of HIV infections averted (*magnitude of impact*) for each age-group targeted intervention. The blue line describes the expansion of the program with minimal diminishing of returns and the red line describes the expansion of the program with considerable diminishing of returns. **B)** Frontier-policy plot classifying the different policy domains based on the theme of maximizing program efficiency (maximizing gain while minimizing cost). Circle size represents the magnitude of the impact. **C)** Frontier-policy plot delineating the different policy domains based on the theme of maximizing the total impact of the VMMC program. Circle size here represents the total number of VMMCs needed relative to the baseline VMMC intervention scenario. In both **B** and **C**, the orange circles represent the age brackets that fall within the optimal policy domain and the blue circle represents the baseline VMMC intervention scenario.

The VMMC program efficiency of age-group prioritization is illustrated in Fig 4. The expansion path in Fig 4A represents the incremental change in total cost of the VMMC program relative to the incremental change in total number of HIV infections averted for each age-group targeted intervention. It starts with targeting the age group that has the highest effectiveness, which is the 20–24 year old (Fig 3A). The program then expanded by adding the other age groups based on a hierarchy of decreasing effectiveness. Though the number of infections averted increased with each age-group expansion, the total cost also increased and at a higher rate. This was especially true as the expansion reached the population older than 35 years of age, or younger than 15 years of age. Here, the diminishing returns of the expansion became evident, highlighting the decline in program efficiency. Fig 4B provides an alternative view of program efficiency through the Gain/Pain index. This measure was >1 only when male populations 15–19, 20–24, 15–24, 15–29, and 15–34 years of age were targeted, affirming the result that this age range brackets the populations with the highest program efficiency. This measure was <1 when targeting male populations 10–14, 25–29, 30–34, 35–39, 40–44, 45–49, 10–24, 10–29, 10–34, and 10–49 years of age.

Fig 4B and 4C show the policy-favorable domains using policy-frontier plots. In Fig 4B, the policy theme is maximizing program efficiency, thereby showing the Gain/Pain index at different scales of the VMMC program. Prioritizing male populations 15–19, 20–24, 15–24, 15–29, and/or 15–34 years of age fit into the optimal policy domain—that is, the domain of favorable program efficiency (Gain/Pain index > 1)—and also favorable total program cost (total program cost being less than that of the baseline intervention scenario of 80% VMMC coverage among the 15–49 year age group). By adding 10–14 year old males to the prioritized age groups (i.e., 10–24, 10–29, 10–34, and/or 10–49 years of age), the VMMC program became unfavorable both in terms of program efficiency (Gain/Pain index < 1) and total cost (total program cost being equal to or higher than that of the baseline intervention scenario). The remaining age-group targeting schemes fit in policy domains that were unfavorable for program efficiency and/or total program cost.

In Fig 4C, the policy theme is maximizing the magnitude of the program’s impact. Thus the figure shows the results of factoring in magnitude of impact (number of HIV infections averted), total VMMC program cost, and cost-effectiveness. Prioritizing male populations ranging in age from 15–34 provided the optimal policy domain: The total program cost was favorable (less than that of the baseline intervention scenario); cost-effectiveness was favorable (more cost-effective than that of the baseline intervention scenario); and—most important for this policy theme—the magnitude of impact was also favorable (total number of infections averted was nearly as large as that of the baseline intervention scenario). Adding the 10–14 year age group to these age brackets did not undermine the favorability of the magnitude of impact nor total program cost. However, it did render cost-effectiveness unfavorable as the cost-effectiveness of this age group was slightly inferior in comparison with that of the baseline scenario. The remaining age-group targeting schemes were unfavorable for magnitude of impact, total program cost, and/or cost-effectiveness.

In this policy-frontier plot (Fig 4C), targeting the 20–24 achieved 61% of the potential magnitude of impact (number of infections averted by 2025) with 62% fewer VMMCs (by 2017), and 50% lower total cost (by 2025; Table 1). Targeting the 15–24 age bracket, achieved 78% of the potential impact (by 2025) with 40% fewer VMMCs (by 2017) and 30% lower total cost (by 2025). Targeting the 15–29 achieved 87% of the potential impact (by 2025) with 29% fewer VMMCs (by 2017) and 22% lower total cost (by 2025). Table 1 summarizes the quantitative results and implications of the different targeting schemes used in generating the program efficiency and policy domain figures for the age-group prioritization.

### Geographic Prioritization

**Fig 5:**
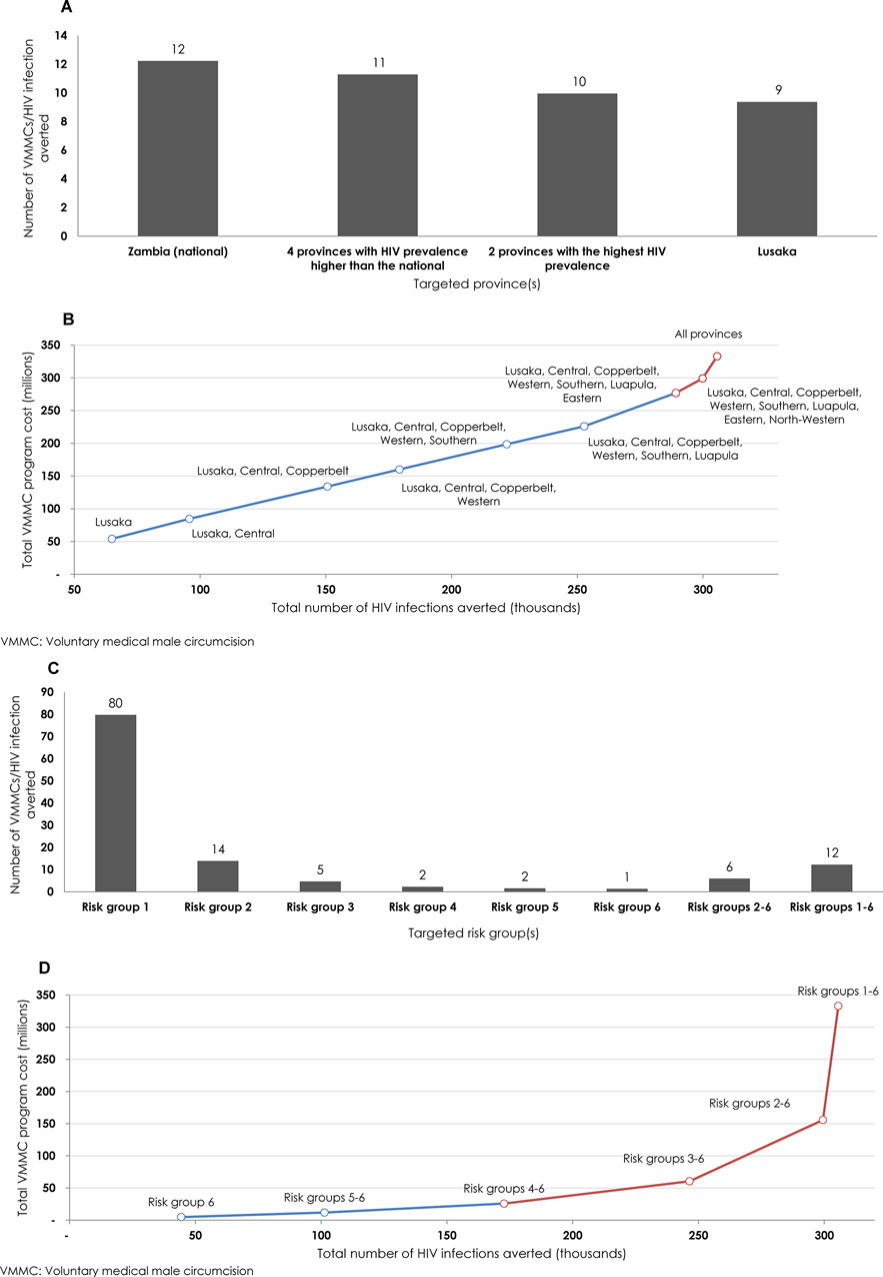
Projected outcomes of geographic and risk-group prioritization. **A)** Number of voluntary medical male circumcisions (VMMCs) needed to avert one HIV infection (*effectiveness*) by 2025 through geographic prioritization. **B)** Expansion path curve showing the incremental increase in total cost of the VMMC program relative to total number of HIV infections averted (*magnitude of impact*) for each geographic targeted intervention. **C)** Number of VMMCs needed to avert one HIV infection by 2025 through risk-group prioritization. **D)** Expansion path curve showing the incremental increase in total cost of the VMMC program relative to total number of HIV infections averted for each risk-group targeted intervention. In both **B** and **D**, the blue line describes the expansion of the program with minimal diminishing of returns and the red line describes the expansion of the program with considerable diminishing of returns.

In the intermediate term, the effectiveness of geographic prioritization ranged from 9 to12 VMMCs per HIV infection averted (Fig 5A). The highest effectiveness was achieved by targeting Lusaka; the province with the highest HIV prevalence in Zambia. The expansion path curve showed the incremental change in total cost of VMMC program relative to the incremental change in total number of HIV infections averted for each geographic area targeted (Fig 5B). The curve was largely linear, with the addition of provinces in a hierarchy of lower HIV prevalence up to provinces whose HIV prevalence was similar to that of Zambia as a whole. Inclusion of provinces with lower HIV prevalence than the national (Eastern, North-Western, and Northern) was reflected as nonlinear growth in total program cost versus total impact, indicating diminishing returns. S4 Table summarizes the quantitative results and implications of the different targeting schemes that served as input to generate the program efficiency assessment for the geographic prioritization.

### Risk-group Prioritization

The effectiveness of risk-group prioritization showed vast variation (Fig 5C). The effectiveness by targeting the different risk groups ranged from one to 80. Targeting males in the highest risk group (Risk group 6) was very effective, with only one VMMC needed per infection averted, while targeting males in the lowest risk group (Risk group 1) required 80 times more VMMCs to avert one HIV infection. In the baseline scenario, where all risk groups were targeted, 12 VMMCs were required to avert one infection. Targeting risk groups 2–6 (i.e., excluding the lowest risk group) was twice as effective as the baseline scenario (6 versus 12 VMMCs per infection averted). The expansion path curve showed the incremental change in total cost of VMMC program relative to the incremental change in total number of HIV infections averted for each risk-group prioritization scheme (Fig 5D). The curve was highly nonlinear with the addition of risk groups, in a hierarchy of lower risk behavior, indicating rapidly diminishing returns with the expansion of the program to populations of lower risk. S5 Table summarizes the quantitative results and implications of the different targeting schemes that were used as input in generating the program efficiency assessment for the risk-group prioritization.

### Sensitivity Analyses

**Fig 6:**
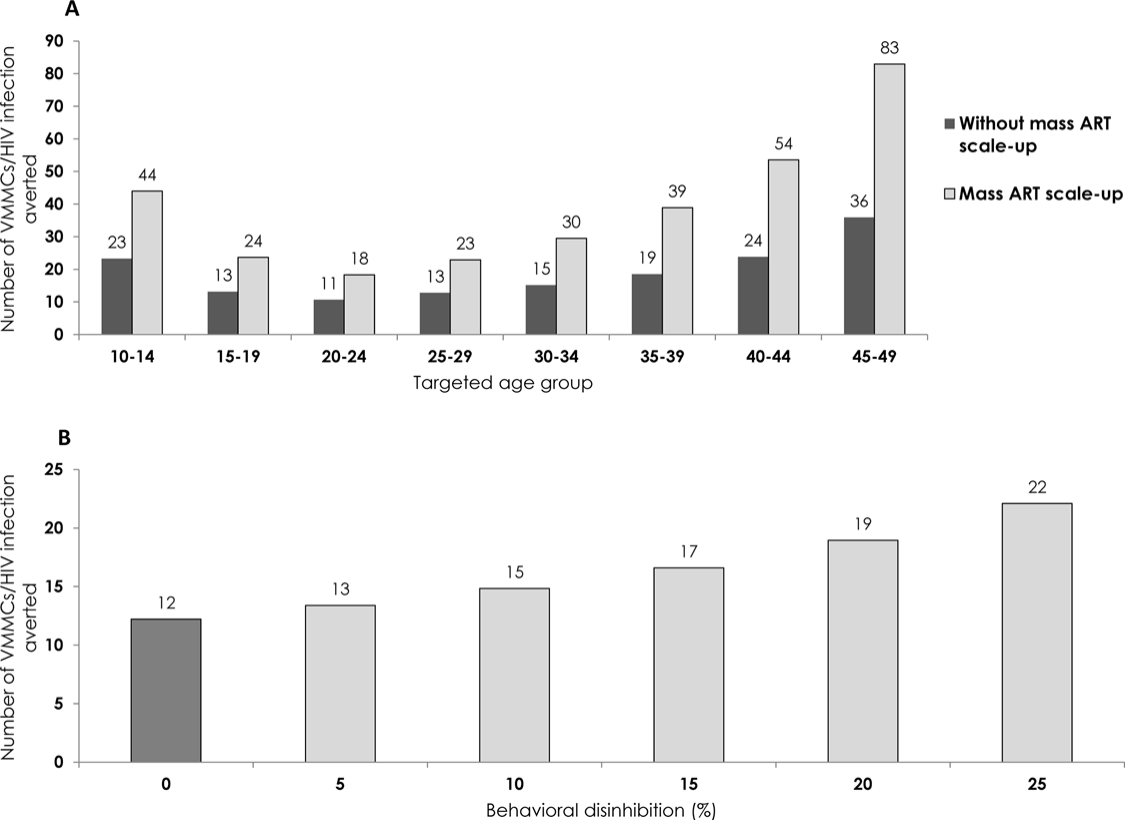
Sensitivity analyses on predictions of intervention outcomes. **A**) Sensitivity of model-predicted intervention effectiveness to antiretroviral therapy (ART) coverage scale-up over the coming decades. The sensitivity analysis compares effectiveness with and without mass ART scale-up for different age-group prioritizations. **B)** Sensitivity of model-predicted intervention effectiveness to sexual risk compensation with VMMC. The sensitivity analysis is conducted by comparing the effectiveness at six different levels of risk compensation, starting with 0% risk compensation, by targeting the 15–49 age bracket.

Fig 6 presents the results of the different conducted sensitivity analyses. Overall, with mass ART scale-up about twice as many VMMCs were needed to avert one HIV infection than without mass ART scale-up (Fig 6A). For instance, the number of VMMCs required by targeting the 20–24 year age group increased from 11 VMMCs per infection averted in the absence of mass ART scale-up to 18 VMMCs per infection averted in the presence of mass ART scale-up. The effectiveness of VMMC was also reduced with risk compensation (Fig 6B). The number of VMMCs needed to avert one infection was almost twice as high if we assume 25% risk compensation, in comparison with the intervention scenario, where no risk compensation was assumed.

## Discussion

Using a novel analytic approach and the most recent program data from Zambia, we demonstrated that program efficiency and public health and economic benefits of VMMC scale-up can be improved by prioritizing young sexually active males and by prioritizing geographic areas where HIV prevalence is considerably higher than the national. Prioritizing by sexual risk group can also markedly improve program efficiency, but the program feasibility of this approach remains to be explored and determined.

Although the success of the current actual VMMC scale-up in Zambia is evident through the accelerated uptake among males year after year since the start of the VMMC initiative in 2007 (Fig 2A), only 61% of the completed VMMCs by the end of 2013 were within the country’s target age group (15–49 year old; S4 Fig). The remaining 39% of circumcised males were young adolescents 10–14 years of age. The age distribution of VMMC uptake is largely influenced by the country’s population pyramid (S5 Fig). For example, although 39% of program-circumcised males were 10–14 year old, they represent only 21% of the 10–14 year old male population in Zambia. Similarly, 15% of circumcised males were 15–19 year old, representing 17% of all 15–19 year old males. Although 40–44 account for only 5% of the population of circumcised males and 45–49 year old 4%, they still represent each 13% of the total population in their respective age groups. Hence, the age distribution of VMMC uptake reflects to a large extent the age distribution of the population pyramid and to a lesser extent differential uptake by age group.

Given the reality of program clients circumcised to date (up to 2014), we estimate that the program would avert 8% fewer HIV infections by 2025 (86,644 compared to 94,369) than would otherwise be the case if all circumcisions had been provided to males within the target age group (15–49 year old). If the total number of circumcisions provided to date had been exclusively among 15–29 year old males, we estimate the program would have averted 51% more HIV infections by 2025 (130,989 compared to 86,644) than would be the case with the current age distribution of VMMCs. These findings not only have important implications for the HIV epidemic, but also provide key considerations for demand generation, service distribution, and communication.

As expected, we found that achieving 1.95 million VMMCs by 2015 is an ambitious target and unlikely to be achieved by any realistic or anticipated scale-up plan (Fig 2A). Therefore, we considered an extended timeframe by which the revised goals could be met: Scenarios with scale-up plans of seven years instead of five. However, adding two more years to the catch-up phase (2016 and 2017) entails performing 580,000 additional VMMCs (cohorts turning 15 years of age during 2016 and 2017) to achieve 80% coverage among males 15–49 years of age by 2017, for a total of 2.53 million VMMCs (based on projected population growth in Zambia, S3 Fig). Even with the addition of these two years to the catch-up phase, achieving the number of VMMCs needed to attain the set target remains unlikely, given the programmatic feasibility challenges related to service availability and demand creation [13]. While the VMMC program in Zambia has nearly doubled its output in one year from 2012 to 2013 (S3 Table), based on the 2014 performance level [48], the program seems to have reached a plateau reflecting increasing funding constraints [13]. There is immediate need to focus the program on achieving greater efficiency by focusing on the most impactful circumcisions.

Our results indicate that the optimal public health benefits of VMMC are dependent on the age at which males undergo circumcision. Prioritizing males ages 15–19, 20–24, or 25–29 year old maximizes the effectiveness of the VMMC program by up to 13%, and maximizes the cost-effectiveness of the VMMC program by up to 19%. However, VMMC impact on the scale of incidence rate reduction through age prioritization depends on the timeframe for which the effect of the VMMC program is assessed (Fig 3C and 3D).

If the program aims to achieve the largest reductions in incidence rate within the catch-up phase (2017), the optimal age groups to prioritize are 15–19 or 20–24 year old. On the other hand, if the program aims to achieve the largest reductions in incidence rate in the longer term (2025 or 2045), then the optimal age groups to prioritize are 10–14, 15–19, or 20–24 year old. These results suggest that the greatest impact of VMMC is achieved by prioritizing the age groups where the risk of infection—that is, the HIV incidence rate—is largest. The highest incidence rate in Zambia was predicted for the 20–24 year age group followed by the 24–29 year age group (S6 Fig). VMMC programs can maximize the public health benefits by reaching males during or immediately prior to the age at which the HIV incidence rate peaks.

Deciding which age groups to prioritize depends on the specific goals of the program and national policy. Decision makers may have different policy priorities, such as program efficiency versus magnitude of impact, versus incidence rate reduction, versus effectiveness, versus cost-effectiveness. The availability of resources, supply and demand models, and on-the-ground country experiences are also key factors that influence policy priorities. Our findings therefore should be considered in conjunction with programmatic feasibility to decide on specific subpopulation prioritization and targets.

To facilitate evidence-informed decision making, we factored epidemiologic and health economic measures collectively to assess optimal policy using program expansion path curves and policy-frontier plots (level two in the assessment framework, Fig 1). The expansion path curves delineated pathways for how VMMC programs can be efficiently expanded to more age groups or subpopulations, by considering total program cost (such as Fig 4A). Our results manifested the diminishing returns of program expansion as males older than 35 years of age or younger than 15 years of age are included. The policy-frontier plots, with both the program efficiency theme and the total impact theme, converge on the optimal age groups to be targeted: 15–24, 15–29 or 15–34 year old (Fig 4B and 4C). Our results suggest that the contribution of males younger than 15 (10–14 year old) and age 35 and older to overall VMMC program efficiency in the short and long terms is limited.

The efficiency of the VMMC program can also be enhanced by prioritizing geographic areas (provinces) where HIV prevalence is considerably higher than the national. VMMC effectiveness can be improved by as much as 23% by prioritizing Lusaka province, which has the highest HIV prevalence in Zambia (Fig 5A). Prioritizing provinces whose HIV prevalence is lower than the national reduces the effectiveness and efficiency of the VMMC program. To achieve the highest program efficiency and maximize magnitude of impact, the VMMC program should be expanded, by prioritizing the provinces with the highest HIV prevalence and low male circumcision prevalence.

Program efficiency with geographic prioritization can be further enhanced by combining age group prioritization with geographic prioritization. For example, VMMC effectiveness can be improved by as much as 29% by prioritizing 20–24 year old males in Lusaka compared to prioritizing 15–49 year old males across the country (8.6 versus 12.2 VMMCs per HIV infection averted). As with age-group prioritization, deciding on which geographic areas to prioritize depends on national policies and goals. Decision makers may be required to balance priorities such as parity in service availability and considerations related to maximum VMMC impact and efficiency.

The efficiency of the VMMC program can be immensely improved by prioritizing males with high sexual-risk behavior. Prioritizing males with the highest sexual-risk behavior in the population would increase VMMC effectiveness by an order of magnitude, while prioritizing males with the lowest sexual-risk behavior would reduce the effectiveness by an order of magnitude (Fig 5C). Even simple targeting by risk behavior can markedly increase program effectiveness. For example, by excluding males in the lowest risk group, 98% of the potential impact (by 2025) of the baseline intervention plan would be achieved with 53% fewer VMMCs (by 2017) and 52% lower total cost (by 2025; S5 Table).

Although prioritization by risk can substantially improve the efficiency of VMMC programs, identifying such populations is a key challenge. Programs may also be viewed unfavorably if communities perceive that VMMC is only for males at high sexual risk, thus stigmatizing clients. Generating unbiased and precise measures of sexual risk behavior at the level of the individual is also challenging, as it relies on self-reported data [55]. It is further difficult to operationalize interventions by risk. One potential consideration is for VMMC programs to develop models by which they could reach clients of sex workers by training sex workers to discuss VMMC with their clients.

Although the primary consequence of scaling up VMMC coverage is to reduce the number of new HIV infections in males, by reducing HIV acquisition the intervention indirectly prevents HIV infections in women as well, as it reduces females’ probabilities over time of encountering HIV-infected male sexual partners (Fig 3D). Our results show that with VMMC scale-up, the HIV incidence rate in women steadily decreased by as much as 20% in 2025 (by prioritizing males ages 20–24). This outcome reflects only indirect effects arising from the reduced circulation of HIV following VMMC scale-up and did not include any biological protective effect of VMMC against male-to-female transmission. The evidence supporting a protective effect against male-to-female transmission is inconclusive to date [56–59], but if we assumed such an effect and factored it in, epidemiologic and economic measures of impact would be even more favorable and incidence reduction among females would be higher [60].

Projections of future HIV incidence are uncertain and subject to unknown factors, such as the scale-up of other HIV interventions. The further into the future that incidence is projected, the greater the degree of uncertainty. Scale-up of other interventions may reduce HIV incidence rate in the population substantially, thereby potentially diminishing the benefits of VMMC programs. However, our sensitivity analysis shows that even with an optimistic ART scale-up scenario (as highlighted by recent WHO projections of reducing HIV incidence by 72%), VMMC will remain an impactful and effective intervention, and the scale-up of ART is not likely to change our results of subpopulation prioritization. Although twice as many VMMCs could be needed to avert one HIV infection under an optimistic ART scale-up scenario, this does not undermine the fact that VMMC will remain a cost-effective intervention for an HIV hyper-endemic setting such as Zambia.

In our main predictions we did not incorporate sexual risk compensation, though the ultimate impact of VMMC on HIV prevention will be determined, in part, by changes in sexual behavior postcircumcision. Although there is some evidence for risk compensation following VMMC [1], the majority of empirical data do not support risk compensation [2, 32, 61]. Regardless, our sensitivity analysis affirmed that even with an increase in sexual risk behavior by as much as 25% following circumcision, risk compensation is not likely to undermine the benefit of VMMC as an effective and cost-effective HIV intervention (Fig 6B).

Another uncertainty lies in estimates of VMMC provision costs. Cost-effectiveness of VMMC with age-group prioritization is dependent on the age-specific unit cost of VMMC. If unit cost were to vary widely by age, it may be equally or more cost-effective to reach a subpopulation of relatively lower epidemiologic priority, for instance. Current evidence is insufficient to determine how VMMC unit costs may vary over time and at scale, and how this variation may affect the age-specific unit cost. Informed by program data [43], we assumed a lower unit cost for younger males (S2 Table). Nonetheless, even with a fixed VMMC unit cost at $95 across all age groups, the overall pattern did not change and the 20–24 age group remained the most cost-effective age group to target (S7 Fig).

Though we used an elaborate mathematical model structure to capture the complexity of HIV dynamics, our results may depend on the type of mathematical model structure used. We assumed a fixed VMMC rate throughout the catch-up phase in all scenarios in our main predictions, but we examined the impact of the VMMC program using variable VMMC rates and the results were virtually invariable (not shown). We assumed that individuals stay in the same sexual risk group during their lifespan. However, as informed by earlier analyses [15], this assumption is not likely to affect our results for a susceptibility reduction intervention such as VMMC provided the relative size of each risk group in the population is invariable.

Moreover, our model is dependent on the availability of representative time-trend data for HIV prevalence, baseline male circumcision prevalence, and demographics such as total population size and future population projections. Limitations in the data input can lead to limitations in the model predictions. Therefore we conducted a multivariate uncertainty analysis on our model predictions to assess the impact of uncertainty in the input parameters (S8 Fig). Though the uncertainty interval for the younger ages (i.e., 10–14 year old) and the older ages (i.e., 45–49 year old) were wider, this analysis affirmed the validity of our predictions for the age-group prioritization. To our knowledge, our model was parameterized by the best available empirical HIV epidemiological data, HIV natural history and transmission parameters, HIV prevalence data, male circumcision data, and demographics.

Finally, this framework for assessing the impact of subpopulation prioritization is not meant to impose country-specific performance targets, but instead to inform national policy and programming, by means of a thorough scientific analysis of the implications of subpopulation prioritization. For instance, country programs may decide to maintain current targets but intensify demand creation for particular subpopulations, to maximize epidemiologic and economic impact. VMMC scale-up strategies should be tailored to each country’s context, realities, and goals, as informed by a detailed analysis of their impact such as in the present study, given that subpopulation prioritization is dependent on the distribution of HIV incidence across age, geography, and risk profile.

## Conclusion

We developed a novel analytic approach, expressed in terms of a mathematical model and a three-tiered conceptual framework, as a planning tool to inform national policy and programming of the optimal public health benefits of VMMC through subpopulation prioritization. The framework consolidates epidemiologic, health economics, program efficiency, and policy measures to facilitate scientifically driven, complex decision making at the national level. The framework was applied using Zambia as a model country to demonstrate the utility of this approach. The framework is being implemented in additional countries, and is available for use in others.

We found that prioritizing males ages 15–29 years improves VMMC program efficiency. Including males 10–14 years of age increases the long-term impact of the program, but is less programmatically efficient. Prioritizing males 35 years of age and older does not add more benefits to the efficiency of VMMC programs.

The efficiency of VMMC programs can also be enhanced by prioritizing geographic areas with considerably higher HIV prevalence than the national prevalence as well as prioritizing individuals with higher sexual-risk behavior. Benefits of VMMC through subpopulation prioritization can also be improved with a combination of age, geographic, and risk-group targeting. However, any policy change based on these results should consider programmatic feasibility and national health policy priorities.

Our study refines the strategic direction set by previous mathematical modeling studies, but it does not contradict the longstanding emphasis on making the comprehensive package of VMMC for HIV prevention services available to all adult males. Services should remain available for all males who seek circumcision; our recommendation is that a subset of the male population become the primary focus of services and demand creation.

## Supporting Information

S1 Fig Comparison of HIV prevalence in each province in Zambia with the national HIV prevalence. (DOCX)

S2 Fig Map of Zambia showing HIV prevalence and the proportion of the country’s total population in each province. (DOCX)

S3 Fig Model prediction of total population size (males and females) in Zambia. (DOCX)

S4 Fig Proportion of total voluntary medical male circumcisions (VMMCs) performed in each five-year age band. (DOCX)

S5 Fig Current age distribution of voluntary medical male circumcision (VMMC) coverage in Zambia. (DOCX)

S6 Fig HIV incidence rate in each five-year age band: Short term (2017) and intermediate term (2025). (DOCX)

S7 Fig Cost of voluntary medical male circumcision (VMMC) per HIV infection averted by 2025 (cost-effectiveness) assuming a fixed age-specific unit cost. (DOCX)

S8 Fig Uncertainty intervals by prioritized age group for the number of voluntary medical male circumcisions (VMMCs) needed to avert one HIV infection by 2025. (DOCX)

S1 Table Model assumptions in terms of parameter values. (DOCX)

S2 Table Age-specific unit cost of voluntary medical male circumcision (VMMC). (DOCX)

S3 Table Current scale-up plan and forecast scenario of the voluntary medical male circumcision (VMMC) program. (DOCX)

S4 Table Epidemic impact of prioritizing different geographic locations in the voluntary medical male circumcision (VMMC) program. (DOCX)

S5 Table Epidemic impact of prioritizing different risk groups in the voluntary medical male circumcision (VMMC) program. (DOCX)

S1 Text Further Details on the Age-Structured Mathematical (ASM) Model. (DOCX)

S2 Text Inclusion of Provinces for Geographic Prioritization. (DOCX)

## Abbreviations

VMMC: voluntary medical male circumcision
ASM: age-structured mathematical
WHO: World Health Organization
UNAIDS: Joint United Nations Programme on HIV/AIDS
DMPPT: Decision-Makers’ Program Planning Tool
SSA: sub-Saharan Africa
USD: U.S. dollars
DHS: Demographic and Health Survey
HSS-ANC: HIV sentinel surveillance-antenatal clinic
ART: antiretroviral therapy
