# Supplementary material for: Investigating Voluntary Medical Male Circumcision Program Efficiency Gains through Subpopulation Prioritization: Insights from Application to Zambia
Source: PLoS One. 2015 Dec 30;10(12):e0145729. doi: 10.1371/journal.pone.0145729 (PMC4696770; doi:10.1371/journal.pone.0145729)
Supplement: S1 Fig — (DOCX) [file pone.0145729.s001.docx]

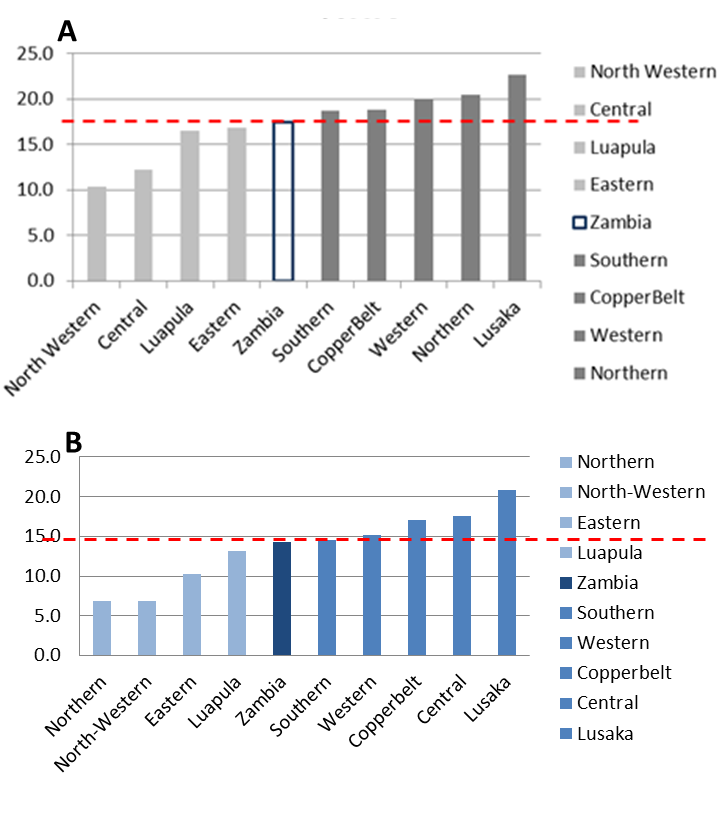


**Fig. S1.** **Comparison of HIV prevalence in each province in Zambia with the national HIV prevalence. A)** based on the 2011 HIV sentinel surveillance from antenatal clinics (HSS-ANC) [1] and **B)** based on the 2007 Demographic and Health Survey (DHS) [2].

**References**

1. Vandament L. Zambia 2011 HIV sentinel surveillance data from antenatal clinics. Country-level Data, Lusaka, Zambia 2013.

2. Zambia Demographic and Health Survey 2007. Available: <http://dhsprogram.com/pubs/pdf/FR211/FR211%5Brevised-05-12-2009%5D.pdf> [Internet]. CSO and Macro International Inc. 2009.
