# Supplementary material for: Investigating Voluntary Medical Male Circumcision Program Efficiency Gains through Subpopulation Prioritization: Insights from Application to Zambia
Source: PLoS One. 2015 Dec 30;10(12):e0145729. doi: 10.1371/journal.pone.0145729 (PMC4696770; doi:10.1371/journal.pone.0145729)
Supplement: S2 Fig — (DOCX) [file pone.0145729.s002.docx]

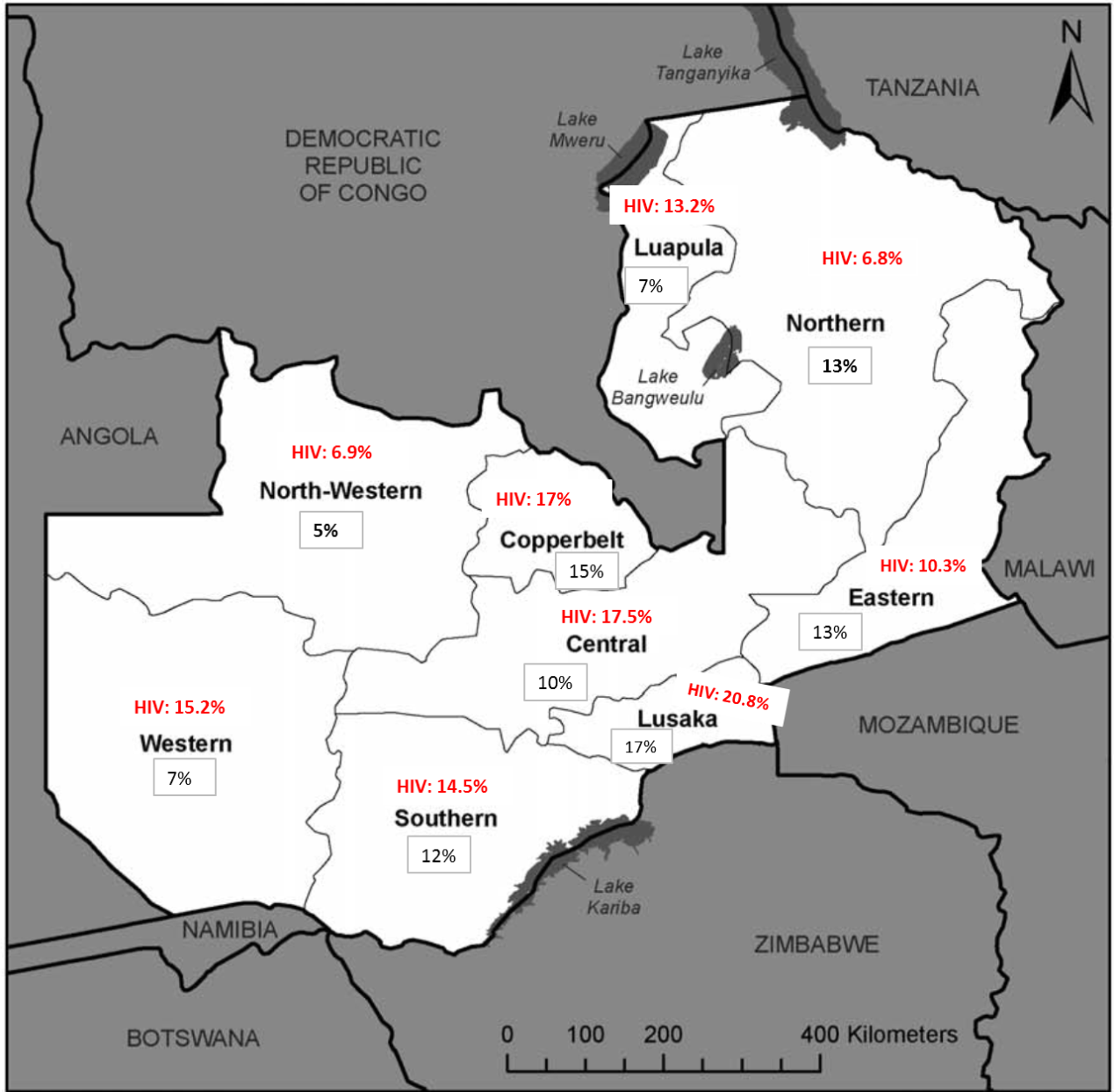


**Fig. S2.** **Map of Zambia showing HIV prevalence^*^ and the proportion of the country’s total population in each province^#^**

Sources: ^*^2007 Demographic and Health Survey (DHS) [1] ^#^2010 Census of Population and Housing [2]

**References**

1. Zambia Demographic and Health Survey 2007. Available: <http://dhsprogram.com/pubs/pdf/FR211/FR211%5Brevised-05-12-2009%5D.pdf> [Internet]. CSO and Macro International Inc. 2009.

2. Central Statistical Office, Republic of Zambia. Zambia 2010 Census of Population and Housing. Available: <http://unstats.un.org/unsd/demographic/sources/census/2010_phc/Zambia/PreliminaryReport.pdf> 2010.
