# Supplementary material for: Investigating Voluntary Medical Male Circumcision Program Efficiency Gains through Subpopulation Prioritization: Insights from Application to Zambia
Source: PLoS One. 2015 Dec 30;10(12):e0145729. doi: 10.1371/journal.pone.0145729 (PMC4696770; doi:10.1371/journal.pone.0145729)
Supplement: S4 Fig — (DOCX) [file pone.0145729.s004.docx]

**
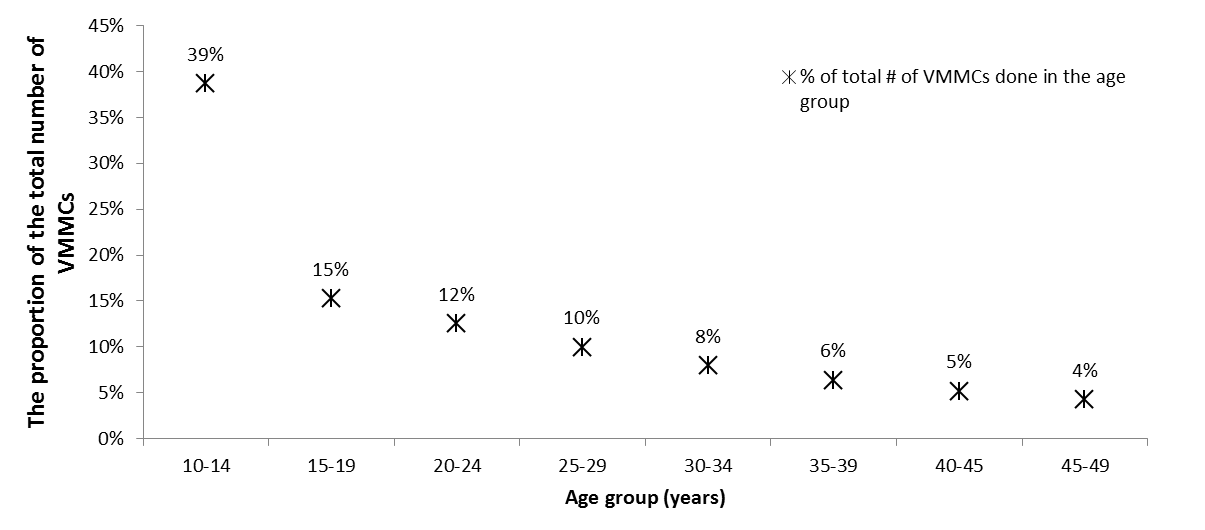
**

**Fig. S4. Proportion of total voluntary medical male circumcisions (VMMCs) performed in each five-year age band**
