# Supplementary material for: Investigating Voluntary Medical Male Circumcision Program Efficiency Gains through Subpopulation Prioritization: Insights from Application to Zambia
Source: PLoS One. 2015 Dec 30;10(12):e0145729. doi: 10.1371/journal.pone.0145729 (PMC4696770; doi:10.1371/journal.pone.0145729)
Supplement: S5 Fig — (DOCX) [file pone.0145729.s005.docx]

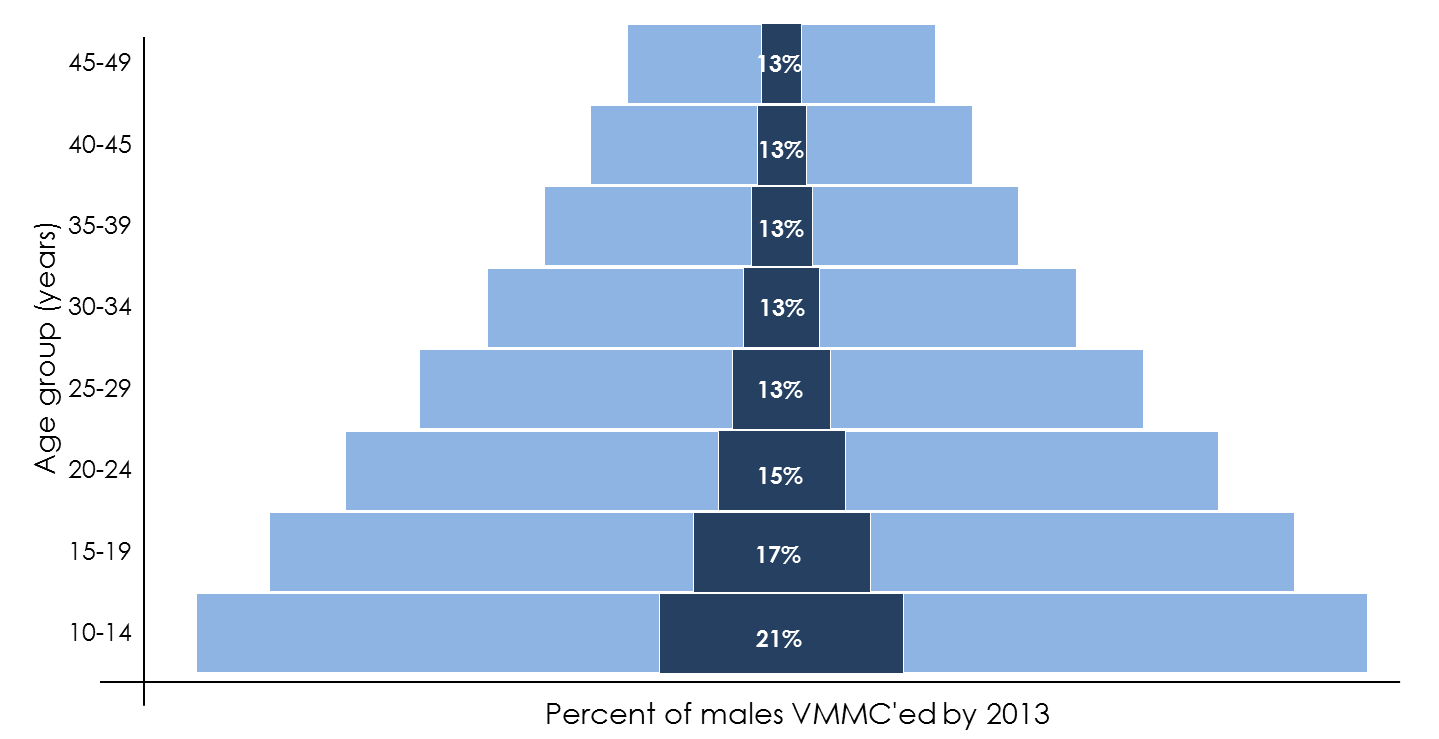


**Fig. S5. Current age distribution of voluntary medical male circumcision (VMMC) coverage in Zambia^*^**

^*^Based on VMMC-program-delivered circumcisions from 2007 through 2013
