# Supplementary figures and images for: Investigating Voluntary Medical Male Circumcision Program Efficiency Gains through Subpopulation Prioritization: Insights from Application to Zambia
Source: PLoS One. 2015 Dec 30;10(12):e0145729. doi: 10.1371/journal.pone.0145729 (PMC4696770; doi:10.1371/journal.pone.0145729)

**
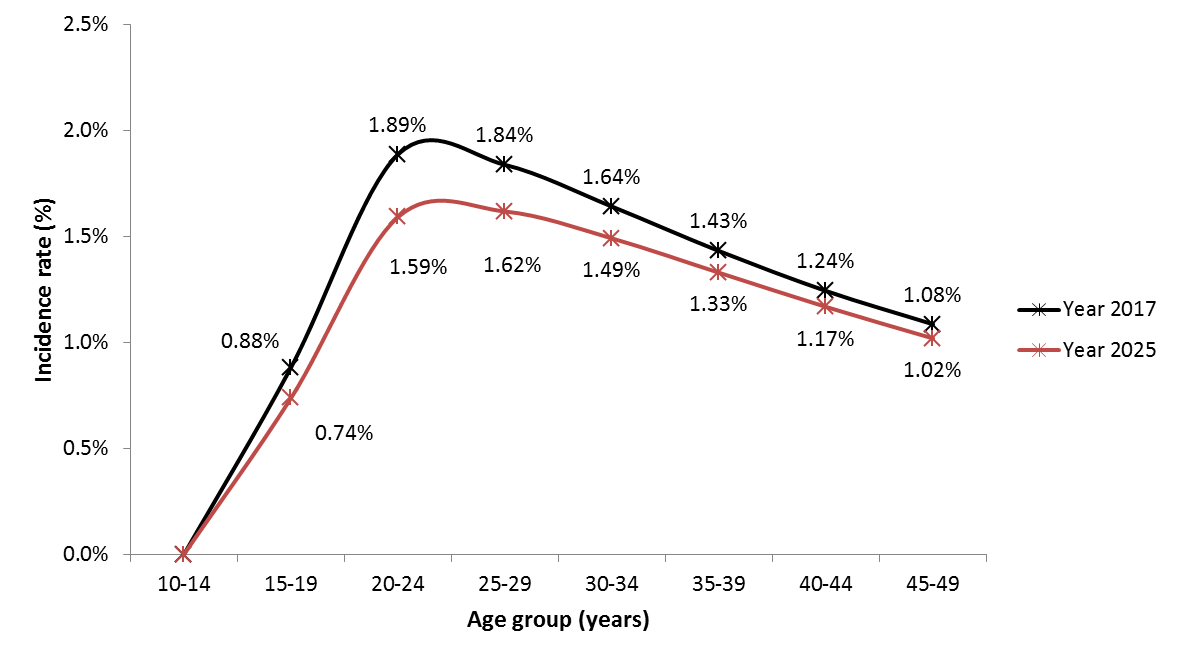
**

**Fig. S6. HIV incidence rate in each five-year age band: Short term (2017) and intermediate term (2025)**

Supplement: S6 Fig — (DOCX) [file pone.0145729.s006.docx]
