# Supplementary material for: Investigating Voluntary Medical Male Circumcision Program Efficiency Gains through Subpopulation Prioritization: Insights from Application to Zambia
Source: PLoS One. 2015 Dec 30;10(12):e0145729. doi: 10.1371/journal.pone.0145729 (PMC4696770; doi:10.1371/journal.pone.0145729)
Supplement: S7 Fig — (DOCX) [file pone.0145729.s007.docx]

Cost-effectiveness ($/HIA)


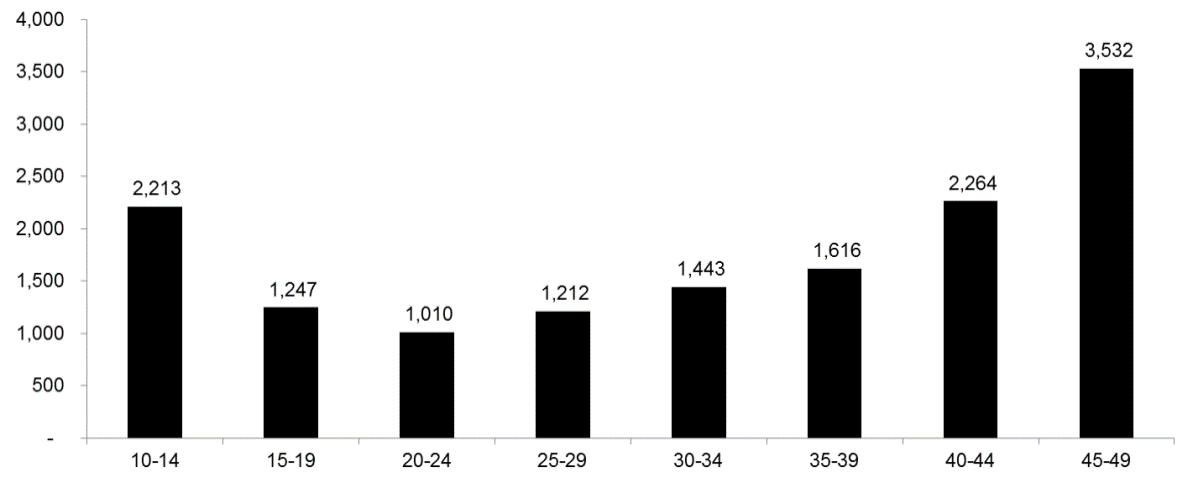


HIA: HIV infection averted

**Fig. S7. Cost of voluntary medical male circumcision (VMMC) per HIV infection averted by 2025 (cost-effectiveness) assuming a fixed age-specific unit cost^*^**

^*^Unit cost of USD$95
