# Supplementary material for: Investigating Voluntary Medical Male Circumcision Program Efficiency Gains through Subpopulation Prioritization: Insights from Application to Zambia
Source: PLoS One. 2015 Dec 30;10(12):e0145729. doi: 10.1371/journal.pone.0145729 (PMC4696770; doi:10.1371/journal.pone.0145729)
Supplement: S8 Fig — (DOCX) [file pone.0145729.s008.docx]

**
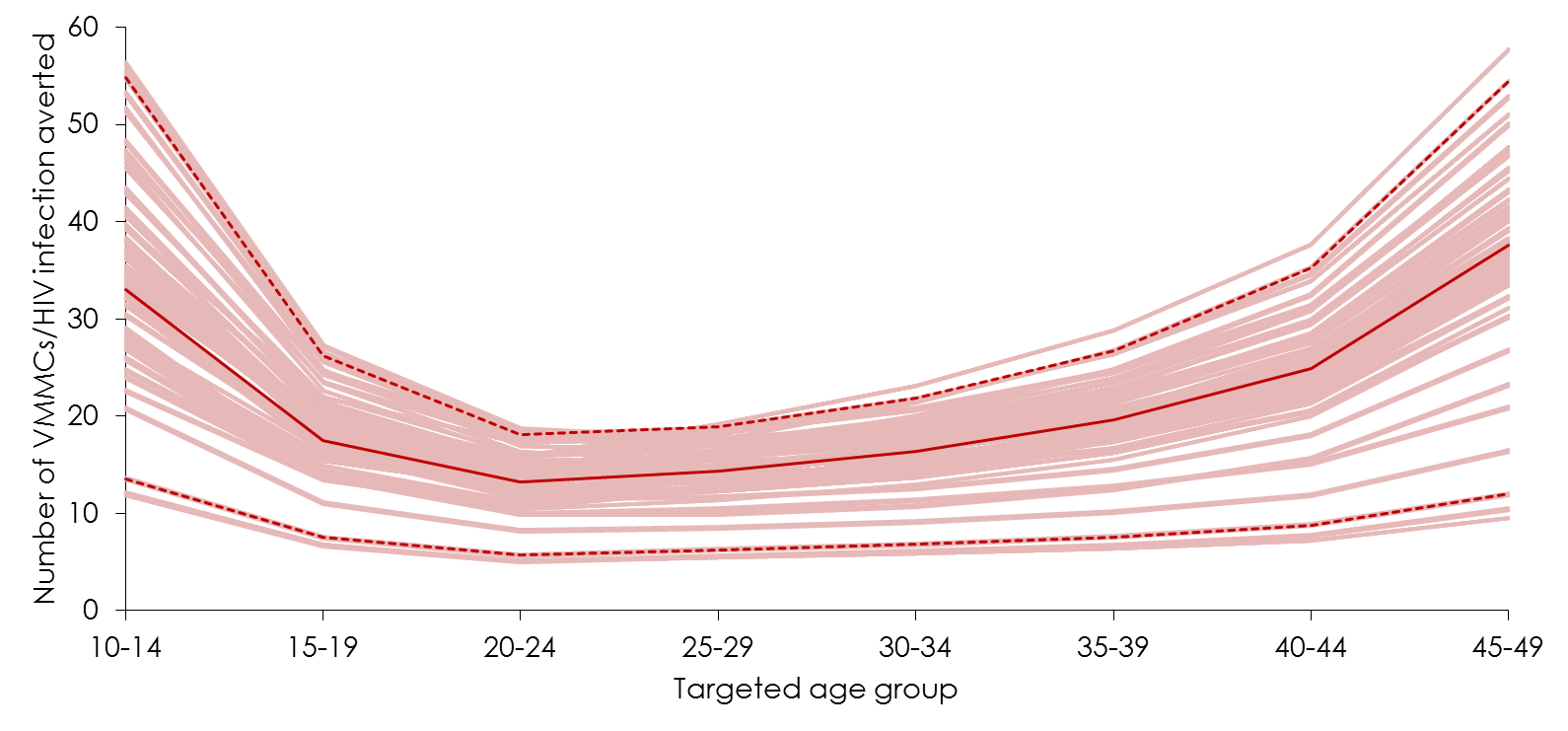
**

**Fig. S8. Uncertainty intervals by prioritized age group for the number of voluntary medical male circumcisions (VMMCs) needed to avert one HIV infection by 2025.** The solid red line represents the geometric mean of model runs, while the dashed lines bracket the 95% uncertainty interval.
