# Supplementary material for: Investigating Voluntary Medical Male Circumcision Program Efficiency Gains through Subpopulation Prioritization: Insights from Application to Zambia
Source: PLoS One. 2015 Dec 30;10(12):e0145729. doi: 10.1371/journal.pone.0145729 (PMC4696770; doi:10.1371/journal.pone.0145729)
Supplement: S1 Table — (DOCX) [file pone.0145729.s009.docx]

**Table S1.** **Model assumptions in terms of parameter values**

| Assumption | Parameter value | Sources |
| --- | --- | --- |
| HIV transmission probability per coital act per stage of infection ($p_{I_{\beta,i}\to S_{j}}^{HIV}$): | | |
| Acute stage | 0.0107 | [1-4] |
| Latent stage | 0.0008 | [4, 5] |
| Late stage | 0.0042 | [1, 2, 4] |
| Duration of each of HIV stages ($1/\omega_{I_{\beta}}$): |  |  |
| Acute stage | 49 days | [1, 4, 6-9] |
| Latent stage | 9.0 years | [1, 4, 6-9] |
| Late stage | 2.0 years | [1, 4, 6-9] |
| Frequency of coital acts per HIV stage ($n_{I_{\beta,i}\leftrightarrow S_{j}}$): |  |  |
| Acute stage | 10.6 per month | [4] |
| Latent stage | 11.0 per month | [4] |
| Late stage | 7.1 per month | [4] |
| Duration of sexual partnerships ($\tau_{I_{\beta,i}\leftrightarrow S_{j}}$): | 6 month | Representative value |
| Degree of assortativeness for age group mixing ($e_{1}$) | 0.7 | Representative value |
| Degree of assortativeness for risk group mixing ($e_{2}$) | 0.3 | [5] |
| The scale parameter in the gamma distribution of the population across the risk groups ($b$) | 1.1 | [10] |
| The exponent parameter in the power law function of the distribution of sexual risk behavior ($\alpha$) | 2.3 | [11, 12] |
| The efficacy of male circumcision (*q*) | 60% | [13-15] |
| Baseline male circumcision ($f_{cm}$) | 12.85% | [16] |

**References**

1. Pinkerton SD. Probability of HIV transmission during acute infection in Rakai, Uganda. AIDS Behav. 2008;12(5):677-84. Epub 2007/12/08. doi: 10.1007/s10461-007-9329-1. PubMed PMID: 18064559.

2. Hollingsworth TD, Anderson RM, Fraser C. HIV-1 transmission, by stage of infection. J Infect Dis. 2008;198(5):687-93. Epub 2008/07/30. doi: 10.1086/590501. PubMed PMID: 18662132.

3. Wawer MJ, Reynolds SJ, Serwadda D, Kigozi G, Kiwanuka N, Gray RH. Might male circumcision be more protective against HIV in the highly exposed? An immunological hypothesis. Aids. 2005;19(18):2181-2. PubMed PMID: 16284475.

4. Wawer MJ, Gray RH, Sewankambo NK, Serwadda D, Li X, Laeyendecker O, et al. Rates of HIV-1 transmission per coital act, by stage of HIV-1 infection, in Rakai, Uganda. J Infect Dis. 2005;191(9):1403-9. Epub 2005/04/06. doi: 10.1086/429411. PubMed PMID: 15809897.

5. Abu-Raddad LJ, Longini IM, Jr. No HIV stage is dominant in driving the HIV epidemic in sub-Saharan Africa. AIDS. 2008;22(9):1055-61. Epub 2008/06/04. doi: 10.1097/QAD.0b013e3282f8af84. PubMed PMID: 18520349.

6. UNAIDS. UNAIDS Reference Group on Estimates, Modelling and Projections. 2007.

7. UNAIDS/WHO. AIDS epidemic update 2007. 2007.

8. UNAIDS/WHO. AIDS epidemic update 2010: UNAIDS fact sheet 2010. Available: <http://www.unaids.org/documents/20101123_FS_SSA_em_en.pdf>.

9. UNAIDS. Epidemiological data, HIV estimates 1990-2013. 2013. Available: <http://www.unaids.org/en/dataanalysis/datatools/aidsinfo>.

10. Cuadros DF, Crowley PH, Augustine B, Stewart SL, Garcia-Ramos G. Effect of variable transmission rate on the dynamics of HIV in sub-Saharan Africa. BMC Infect Dis. 2011;11:216. Epub 2011/08/13. doi: 10.1186/1471-2334-11-216. PubMed PMID: 21834977; PubMed Central PMCID: PMC3175213.

11. Awad SF, Cuadros DF, Abu-Raddad LJ. Generic patterns of HIV infection distribution in human populations. Under preparation. 2012.

12. Liljeros F, Edling CR, Amaral LAN, Stanley HE, Åberg Y. The web of human sexual contacts. Promiscuous individuals are the vulnerable nodes to target in safe-sex campaigns.2001; 411.

13. Auvert B, Taljaard D, Lagarde E, Sobngwi-Tambekou J, Sitta R, Puren A. Randomized, controlled intervention trial of male circumcision for reduction of HIV infection risk: the ANRS 1265 Trial. PLoS Med. 2005;2(11):e298. PubMed PMID: 16231970.

14. Bailey RC, Moses S, Parker CB, Agot K, Maclean I, Krieger JN, et al. Male circumcision for HIV prevention in young men in Kisumu, Kenya: a randomised controlled trial. Lancet. 2007;369(9562):643-56. Epub 2007/02/27. doi: 10.1016/S0140-6736(07)60312-2. PubMed PMID: 17321310.

15. Gray RH, Kigozi G, Serwadda D, Makumbi F, Watya S, Nalugoda F, et al. Male circumcision for HIV prevention in men in Rakai, Uganda: a randomised trial. Lancet. 2007;369(9562):657-66. Epub 2007/02/27. doi: 10.1016/S0140-6736(07)60313-4. PubMed PMID: 17321311.

16. Zambia Demographic and Health Survey 2007. Available: <http://dhsprogram.com/pubs/pdf/FR211/FR211%5Brevised-05-12-2009%5D.pdf> [Internet]. CSO and Macro International Inc. 2009.
