# Supplementary material for: Investigating Voluntary Medical Male Circumcision Program Efficiency Gains through Subpopulation Prioritization: Insights from Application to Zambia
Source: PLoS One. 2015 Dec 30;10(12):e0145729. doi: 10.1371/journal.pone.0145729 (PMC4696770; doi:10.1371/journal.pone.0145729)
Supplement: S2 Table — (DOCX) [file pone.0145729.s010.docx]

**Table S2. Age-specific unit cost of voluntary medical male circumcision (VMMC)**

| Cost structure | Age band | Unit cost of VMMC ($ USD) |
| --- | --- | --- |
| Fixed unit cost structure | 0-49 | 95 |
| Disaggregated unit cost structure | 0-4 | 30 |
|  | 5-9 | 90.25 |
|  | 10-14 | 90.25 |
|  | 15-19 | 95 |
|  | 20-24 | 99.75 |
|  | 25-29 | 104.74 |
|  | 30-34 | 109.97 |
|  | 35-39 | 109.97 |
|  | 40-44 | 109.97 |
|  | 45-49 | 109.97 |

The differential cost structure of VMMC that was used in the model. The cost structure is based on VMMC program data [1]. The base year for the US dollars was 2011.

**References**

1. Vandament L. Program circumcision unit cost per actual VMMC program data from Zambia. Country-level data, Lusaka, Zambia 2013.
