# Supplementary material for: Investigating Voluntary Medical Male Circumcision Program Efficiency Gains through Subpopulation Prioritization: Insights from Application to Zambia
Source: PLoS One. 2015 Dec 30;10(12):e0145729. doi: 10.1371/journal.pone.0145729 (PMC4696770; doi:10.1371/journal.pone.0145729)
Supplement: S3 Table — (DOCX) [file pone.0145729.s011.docx]

**Table S3. Current scale-up plan and forecast scenario of the voluntary medical male circumcision (VMMC) program**

| Scale-up scenario |  | Total number of VMMCs per year | | | | | | | |
| --- | --- | --- | --- | --- | --- | --- | --- | --- | --- |
|  | **2007** | **2008** | **2009** | **2010** | **2011** | **2012** | **2013** | **2014** | **2015** |
| Original plan of the Zambia VMMC scale-up program | - | - | - | - | 84,604^*^ | 198,511^*^ | 270,528^*^ | 526,818^*^ | 868,538^*^ |
| Forecast plan based on no-growth after 2013 | 304^#^ | 2,454^#^ | 17,180^#^ | 61,911^#^ | 85,151^#^ | 164,082^#^ | 294,466^#^ | 294,466^*^ | 294,466^*^ |
| Forecast plan based on current VMMC program | 304^#^ | 2,454^#^ | 17,180^#^ | 61,911^#^ | 85,151^#^ | 164,082^#^ | 294,466^#^ | 323,950^*^ | 402,531^*^ |

The yearly number of VMMCs in each of the different scale-up scenarios is examined to assess the feasibility of the original Zambia VMMC scale-up plan.

^*^ Predicted number of VMMCs. ^#^ Achieved number of VMMCs.
VMMC: Voluntary medical male circumcision.
