# Supplementary material for: Investigating Voluntary Medical Male Circumcision Program Efficiency Gains through Subpopulation Prioritization: Insights from Application to Zambia
Source: PLoS One. 2015 Dec 30;10(12):e0145729. doi: 10.1371/journal.pone.0145729 (PMC4696770; doi:10.1371/journal.pone.0145729)
Supplement: S4 Table — (DOCX) [file pone.0145729.s012.docx]

**Table S4. Epidemic impact of prioritizing different geographic locations in the voluntary medical male circumcision (VMMC) program**

| Area  (15-49 year old) | #VMMC/HIA  (2010-25) | #VMMCs  (2010-17) (millions) | Ad MCs (2018-25)  (millions) | HIA  (2010-17) (millions) | HIA  (2010-25) (millions) | Cost/HIA  ($ USD)  (2010-25) | Total cost (billion)  (2010-25) |
| --- | --- | --- | --- | --- | --- | --- | --- |
| **Zambia** | **12** | **2.53** | **1.2** | **0.13** | **0.31** | **1,089** | **0.33** |
| The four provinces with highest HIV prevalence | 11 | 1.34 (53%) | 0.66 | 0.07 (60%) | 0.18 (58%) | 1,001 (91%) | 0.18 (53%) |
| The two provinces with highest HIV prevalence | 10 | 0.65 (25%) | 0.31 | 0.04 (34%) | 0.10 (31%) | 885 (81%) | 0.08 (25%) |
| Lusaka | 9 | 0.41 (16%) | 0.19 | 0.03 (24%) | 0.06 (21%) | 833 (76%) | 0.005 (16%) |

The number of VMMCs needed to avert one HIV infection (2010–2025) (*effectiveness*); the total number of VMMCs needed to reach 80% coverage by 2017; the additional number of VMMCs needed during the sustainability phase (2018–2025); the total number of HIV infections averted (2010–2017 and 2010–2025) (*magnitude of impact*); the cost needed to avert one HIV infection (2010–2025) (*cost-effectiveness*); and the total program cost (2010–2025) (*program cost*). Targeting the 15–49 year old male population in the whole of Zambia is used as the baseline VMMC intervention scenario for comparison purposes. The numbers in parentheses indicate the fractions achieved relative to the baseline.

VMMC: Voluntary medical male circumcision, HIA: HIV infection(s) averted
