# Supplementary material for: Investigating Voluntary Medical Male Circumcision Program Efficiency Gains through Subpopulation Prioritization: Insights from Application to Zambia
Source: PLoS One. 2015 Dec 30;10(12):e0145729. doi: 10.1371/journal.pone.0145729 (PMC4696770; doi:10.1371/journal.pone.0145729)
Supplement: S5 Table — (DOCX) [file pone.0145729.s013.docx]

**Table S5. Epidemic impact of prioritizing different risk groups in the voluntary medical male circumcision (VMMC) program**

| Risk group (15-49 year old) | #VMMC/HIA  (2010-25) | #VMMCs  (millions) (2010-17) | Additional VMMCs (2018-25) | HIA  (millions) (2010-25) | Cost/HIA  ($ USD)  (2010-25) | Total cost (billion)  (2010-25) |
| --- | --- | --- | --- | --- | --- | --- |
| **1-6** | **12** | **2.53** | **1.2** | **0.31** | **1,089** | **0.33** |
| 1 | 80 | 1.33 (52%) | 0.64 | 0.02 (8%) | 7,145 (656%) | 0.18 (53%) |
| 2 | 14 | 0.72 (29%) | 0.34 | 0.08 (25%) | 1,234 (113%) | 0.09 (29%) |
| 3 | 5 | 0.27 (11%) | 0.13 | 0.09 (28%) | 403 (37%) | 0.04 (10%) |
| 4 | 2 | 0.11 (4%) | 0.05 | 0.07 (254 | 194 (18%) | 0.01 (4%) |
| 5 | 2 | 0.05 (2%) | 0.03 | 0.06 (18%) | 129 (12%) | 0.01 (2%) |
| 6 | 1 | 0.03 (1%) | 0.02 | 0.04 (15%) | 108 (10%) | 0.01 (1%) |
| 2-6 | 6 | 1.19 (47%) | 0.57 | 0.30 (98%) | 520 (48%) | 0.26 (48%) |

The number of VMMCs needed to avert one HIV infection (2010–2025) (*effectiveness*); the total number of VMMCs needed to reach 80% coverage by 2017; the additional number of VMMCs needed during the sustainability phase (2018-2025); the total number of HIV infections averted (2010–2025) (*magnitude of impact*); the cost needed to avert one HIV infection (2010–2025) (*cost-effectiveness*); and the total program cost (2010–2025) (*program cost*). The total 15–49 year old male population is used as the baseline VMMC intervention scenario for comparison purposes. The numbers in parentheses indicate the fractions achieved relative to the baseline.

VMMC: Voluntary medical male circumcision, HIA: HIV infection(s) averted
