# Supplementary material for: Investigating Voluntary Medical Male Circumcision Program Efficiency Gains through Subpopulation Prioritization: Insights from Application to Zambia
Source: PLoS One. 2015 Dec 30;10(12):e0145729. doi: 10.1371/journal.pone.0145729 (PMC4696770; doi:10.1371/journal.pone.0145729)
Supplement: S1 Text — (DOCX) [file pone.0145729.s014.docx]

**Text S1**

**Further Details on the Age-Structured Mathematical (ASM) Model**

**Model Structure**

A deterministic compartmental model of the heterosexual transmission of HIV was constructed. The model was developed based on extension of earlier models [1-5]. The model stratifies the population into compartments according to sex, circumcision status, HIV status and stage of infection, sexual risk-activity group, and age group, using sets of coupled nonlinear ordinary differential equations, each set of which is for a specific risk and age group.

Females:

Non-circumcised males:

Circumcised males:

To accommodate the heterogeneity in sexual risk behavior, we stratified the population into six sexual risk groups, defined with the index $i$ ($i=1,2...6$) representing the low to higher risk groups. The index $a$ represents the age cohort ($a=1,2,...,20$) in the population. The population is stratified into three groups based on sex and male circumcision status: females, noncircumcised males, and circumcised males (subscripts$f$, $nm$, and $cm$, respectively). Here, $S_{y}(a,i)$ is the HIV-susceptible population belonging to sex and circumcision group $y$. $I_{\alpha,y}(a,i)$ are the populations with HIV where the index $\alpha$ marks the stage of HIV pathogenesis; $\alpha=1,2,3$ stand for acute, latent, and advanced stages, respectively. $N_{f}(1,i)$ and $N_{m}(1,i)$ are the female and male population sizes of each $i$-risk group. is the population growth rate parameter, while $f_{cm}$ is the fraction of the male population that is circumcised before starting sexual activity (baseline circumcision).

The rate of progression from one HIV stage to the next is described by $\omega_{1}$ (from acute to latent stage) and $\omega_{2}$ (from latent to late stage), while $\omega_{3}$ is the rate of AIDS disease mortality. $\mu_{y}(a)$ is the natural death rate, while $\eta(a)$ is the transition rate from one age group to the next age group. $\gamma(a)$ denotes the average rate at which noncircumcised males are being circumcised as part of a voluntary medical male circumcision (VMMC) intervention. The rate $\Lambda_{y}(a,i)$is the HIV force of infection (hazard rate of infection) experienced by each $S_{y}(a,i)$susceptible population:$\Lambda_{cm}(a,i)=(1-q)(1+r)\Lambda_{nm}(a,i)$

In these expressions, $\rho_{X_{y}(a,i)}$ describes the *effective* new sexual partner change rate for each population variable $X_{y}(a,i)$ (further discussion below).

The parameter $r\in\left[0,\infty\right)$ models the relative increase in the effective rate of partner change due to risk compensation experienced by circumcised males following circumcision. The parameter $q\in\left[0,1\right]$ measures the efficacy of male circumcision against HIV acquisition [6-8]. At the extremes, $q=0$ implies no protection against HIV and $q=1$ implies total protection against HIV.

The parameter $t_{I_{\alpha,y}(b,j)\to S_{y'}(a,i)}$ defines HIV transmission probability per heterosexual partnership between a member of the susceptible population $S_{y'}(a,i)$ and a member of the population with HIV $I_{\alpha,y}(b,j)$:

$$t_{I_{\alpha,y}(b,j)\to S_{y'}(a,i)}=1-\left(1-p_{I_{\alpha,y}(b,j)\to S_{\gamma'}(a,i)}^{HIV}\right)^{n_{I_{\alpha,y}(b,j)\leftrightarrow S_{y'}(a,i)}\tau_{I_{\alpha,y}(b,j)\leftrightarrow S_{y'}(a,i)}}$$

The HIV transmission probability per partnership is expressed in terms of HIV transmission probability per coital act per HIV stage in this partnership ($p_{I_{\alpha,y}(b,j)\to S_{y'}(a,i)}^{HIV}$), the frequency of coital acts per unit time in this partnership ($n_{I_{\alpha,y}(b,j)\leftrightarrow S_{y'}(a,i)}$), and the duration ($\tau_{I_{\alpha,y}(b,j)\leftrightarrow S_{y'}(a,i)}$) of this partnership.

The transmission probability per coital act from an HIV-positive circumcised male to a susceptible female is given by:

$$p_{I_{\alpha,cm}(b,j)\to S_{f}(a,i)}^{HIV}=(1-g)\times p_{I_{\alpha,nm}(b,j)\to S_{f}(a,i)}^{HIV}$$

Here $g$ is a reduction factor in the transmission probability per coital act from an HIV-positive circumcised male to a susceptible female relative to the transmission probability per coital act from an HIV-positive noncircumcised male to a susceptible female.

The mixing among the different risk groups, between females and males, is dictated by the sexual-mixing matrix $G_{y\to y'}(i,j)$, and the mixing among the different age groups, between females and males, is dictated by the age-mixing matrix $H_{y\to y'}(a,b)$. These two matrices provide the probability that an individual of sex $y$ in risk group $i$ and age group $a$ would choose a partner of the opposite sex $y'$ in risk group $j$ and age group $b$ [3]. The two mixing matrices are given by the expressions

$$H_{m\to f}(a,b)=e_{1}\delta_{a,b}+\left(1-e_{1}\right)\frac{\rho_{S_{nm}(b,j)}S_{nm}(b,j)+(1+r)\rho_{S_{nm}(b,k)}S_{cm}(b,j)+\sum_{\alpha=1,2,3} \left(\rho_{I_{\alpha,nm}(b,j)}I_{\alpha,nm}(b,j)+(1+r)\rho_{I_{\alpha,nm}(b,j)}I_{\alpha,cm}(b,j)\right)}{\sum_{h=1}^{n_{2}} \left(\rho_{S_{nm}(b,h)}S_{nm}(b,h)+(1+r)\rho_{S_{nm}(b,h)}S_{cm}(b,h)+\sum_{\alpha=1,2,3} \left(\rho_{I_{\alpha,nm}(b,h)}I_{\alpha,nm}(b,h)+(1+r)\rho_{I_{\alpha,nm}(b,h)}I_{\alpha,cm}(b,h)\right)\right)}$$

$$G_{m\to f}(i,j)=e_{2}\delta_{i,j}+\left(1-e_{2}\right)\frac{\sum_{c=1}^{n_{1}} \left(\rho_{S_{nm}(c,j)}S_{nm}(c,j)+(1+r)\rho_{S_{nm}(c,j)}S_{cm}(c,j)+\sum_{\alpha=1,2,3} \left(\rho_{I_{\alpha,nm}(c,j)}I_{\alpha,nm}(c,j)+(1+r)\rho_{I_{\alpha,nm}(c,j)}I_{\alpha,cm}(c,j)\right)\right)}{\sum_{c=1}^{n_{1}} \sum_{h=1}^{n_{2}} \left(\rho_{S_{nm}(c,h)}S_{nm}(c,h)+(1+r)\rho_{S_{nm}(c,h)}S_{cm}(c,h)+\sum_{\alpha=1,2,3} \left(\rho_{I_{\alpha,nm}(c,h)}I_{\alpha,nm}(c,h)+(1+r)\rho_{I_{\alpha,nm}(c,h)}I_{\alpha,cm}(c,h)\right)\right)}$$

$$H_{f\to m}(a,b)=e'_{1}\delta_{a,b}+\left(1-e'_{1}\right)\frac{\rho_{S_{f}(b,j)}S_{f}(b,j)+\sum_{\alpha=1,2,3} \left(\rho_{I_{\alpha,f}(b,j)}I_{\alpha,f}(b,j)\right)}{\sum_{c=1}^{n_{1}} \sum_{h=1}^{n_{2}} \left(\rho_{S_{f}(c,h)}S_{f}(c,h)+\sum_{\alpha=1,2,3} \left(\rho_{I_{\alpha,f}(c,h)}I_{\alpha,f}(c,h)\right)\right)}$$

$$G_{f\to m}(i,j)=e'_{2}\delta_{i,j}+\left(1-e'_{2}\right)\frac{\sum_{c=1}^{n_{1}} \left(\rho_{S_{f}(c,j)}S_{f}(c,j)+\sum_{\alpha=1,2,3} \left(\rho_{I_{\alpha,f}(c,j)}I_{\alpha,f}(c,j)\right)\right)}{\sum_{c=1}^{n_{1}} \sum_{k=1}^{n_{2}} \left(\rho_{S_{f}(c,k)}S_{f}(c,k)+\sum_{\alpha=1,2,3} \left(\rho_{I_{\alpha,f}(c,k)}I_{\alpha,f}(c,k)\right)\right)}$$

Here, $\delta_{a,b}$ (and $\delta_{i,j}$) is the identity matrix and the parameters $e_{1}, e_{2}\text{, }e_{1}\text{'}$, and $e_{2}\text{'}\in\left[0,1\right]$ measure the degree of assortativeness in the mixing. At the extreme $e_{1}=e_{2}=e_{1}\text{'}=e_{2}\text{'}=0$, the mixing is fully proportional, while at the other extreme $e_{1}=e_{2}=e_{1}\text{'}=e_{2}\text{'}=1$ the mixing is fully assortative, as individuals choose partners only from within their risk and age groups. Once the mixing matrices of one sex are determined (say, males), the other sex mixing matrices are determined through balance of partnerships (see below).

**Sexual Risk Behavior**

*Distribution of sexual risk behavior in the population*

The sexually active population was stratified into a number of risk groups where the proportion of the population initially in each risk group $i$ was determined using a gamma distribution

$$p_{y}(i)=\frac{1}{\vartheta^{k}\Gamma(k)}i^{k-1}e^{\frac{-i}{\vartheta}}.$$

The gamma distribution is motivated by the degree distribution of the number of sexual partners as identified empirically in studies in sub-Saharan Africa [9-12]. Here $k$ is the shape parameter determined through normalization of the distribution, and $\vartheta$ is the scale parameter in the gamma distribution of the population size across the risk groups [9].

*The effective new sexual partner change rate*

The $\rho_{X_{y}(a,i)}$ parameter describes the number of new sexual partners an individual in a specified risk group acquires, but also effectively other behavioral factors that enhance the risk of exposure to the infection such as concurrency and clustering within sexual networks [4, 13-17], sexually transmitted infection cofactor effects [18], as well as variability in sexual risk behavior in the population [19]. Since the exact nature of sexual behavior and sexual networks in sub-Saharan Africa is not well-understood, and varies within and across communities [20, 21], $\rho_{X_{y}(a,i)}$ is effectively a summary measure of the population-specific level of sexual risk behavior, and captures the distribution and strength of the risk of exposure to HIV infection. The form of the $\rho_{X_{y}(a,i)}$ distribution across different risk groups was defined through a power law function as

$\rho_{X_{y}(a,i)}=Ci^{\beta}$.

This form is motivated by simulations using an individual-based network model developed to explore the diversity in the level of sexual risk behavior [22], and also by analyses of empirical sexual networks [23], by analyses of the architecture of complex weighted networks [24, 25], and by an analysis of the average separation between individuals in a network or a subnetwork [26, 27]. The latter can be seen as a proxy of the size of the “ecology” through which an individual can acquire HIV infection. Here $C$ is a constant determined by the average risk behavior and $\beta$ is the exponent parameter that determines the level of variability in the effective sexual partner change rate [22].

*Temporal variation in sexual risk behavior*

Given the evidence for rapidly declining HIV incidence in sub-Saharan Africa [28-30], we incorporated in our model temporal changes in sexual risk behavior. We parameterized the temporal variation (time dependence of $\rho_{X_{y}(a,i)}$) through a Wood-Saxon function [31, 32]. This function is mathematically designed to describe and characterize transitions. It parameterizes any given transition in terms of its scale or strength, smoothness or abruptness, thickness (duration), and the turning point [31, 32]. Using the Wood-Saxon parameterization, $\rho_{X_{y}(a,i)}(t)$is given by:

$\rho_{X_{y}(a,i)}(t)=\rho_{X_{y}(a,i)}\left(1+\frac{Z}{1+\exp\left[\left(t-\xi_{Turning}\right)/\xi_{Duration}\right]}\right)$ .

Here, $\rho_{X_{y}(a,i)}$ is the asymptotic value of $\rho_{X_{y}(a,i)}(t)$ that describes the level of risk behavior well after the transition. $\xi_{Duration}$ describes the transition duration parameter, with the actual duration of the transition given by $\tau=4.4\xi_{Duration}$ (where the effective partner change rate falls from 90% to 10% of its initial value) [31]. Meanwhile, $\xi_{Turning}$ is the turning point year at which the effective partner change rate crosses half the way towards its asymptotic value of $\rho_{X_{y}(a,i)}$.

The level of sexual risk behavior changes during the transition from $\rho_{X_{y}(a,i)}\left(1+Z\right)$ before the transition to $\rho_{X_{y}(a,i)}$ after the transition. Accordingly, the reduction in the level of sexual risk behavior is given by $l=\frac{Z}{1+Z}$.

*Balancing of sexual partnerships between males and females*

A balance in sexual partnerships must be enforced so that partnerships formed by females in any specific subpopulation (risk group and age group) with males in another subpopulation must equal the number of partnerships formed by males in this latter subpopulation with the females in the former subpopulation. Explicitly:

We used a prescription introduced by Garnett and Anderson to balance the partnerships [2]. Thus the rate of sexual partner change rate depends on the partners’ risk group and age group according to [2]:

Here, $B(a,i,b,j)$ is the weight necessary to balance the partnerships between any two subpopulations of females and males. The adjusted partner change rates for females and males are given by:

where $N_{f}(a,i)$ represents the total number of females in age group $a$ and risk group $i$:

$$N_{f}(i)=\left(S_{f}(a,i)+\sum_{\alpha=1}^{3} I_{\alpha,f}(a,i)\right)$$

and $N_{m}(b,j)$ represents the total number of males in age group $b$ and risk group$j$:

$$N_{m}(j)=\left(S_{nm}(j)+\left(1+r\right)S_{cm}(j)+\sum_{\alpha=1}^{3} \left(I_{\alpha,nm}(b,j)+\left(1+r\right)I_{\alpha,cm}(b,j)\right)\right)$$

Here, $\theta_{1}$ is the degree to which females and males “alter” their sexual risk behavior to insure the balance of partnerships.

**Parameter values**

The parameters of the model were derived using the best available empirical data on HIV epidemiology and natural history from sub-Saharan Africa, and are listed in Table S1 along with their references. We based the HIV transmission probability per coital act during each HIV stage on recent re-analyses of the Rakai Study data [4, 33-35]. The frequency of coital acts per each stage of infection was based on the measurements of Wawer et al. [35].

The durations of the acute, latent, and advanced stages were assumed to be 49 days (acute), 9 years (latent), and 2 years (advanced). These choices were based on data compiled by UNAIDS indicating that the average duration from HIV acquisition to death, in the absence of antiretroviral therapy, is about 11 years [36, 37]. These choices were also based on Wawer et al.’s classification [35], a re-analysis of the Rakai data for acute infection [33], and the measured time from seroconversion to death in several cohort studies [28, 38]. The natural mortality rate ($\mu(a)$) is an age-dependent parameter that is determined by the country-specific average life expectancy and survival curve.

The degree of assortativeness ($e_{1}$) for the age mixing was fixed at 0.7, but with a differential age mixing where males from a specific 5-years age group will preferentially mix with females in the 5-years age group below their age group (younger females). As for the parameters of sexual risk behavior, the degree of assortativeness ($e_{2}$) was fixed at 0.3—a representative value based on model calibration of the HIV epidemic in Kisumu, Kenya [4]. Meanwhile, the scale parameter in the gamma distribution of the population across the different risk groups ($b$) was fixed at 1.1, based on fitting empirical data of the degree distribution (number of sexual partners per year) [9]. The exponent parameter in the power law function of the distribution of sexual risk behavior ($\alpha$) was fixed at 2.3, based on analyses of sexual networks and on fitting the distribution of the clustering coefficient of all possible configurations in a sexual network [22, 23]. The constant $C$ (in the power law function) was determined by fitting HIV prevalence data for each country.

The protective effect of male circumcision was parameterized through a reduction of 60% in the force of infection for circumcised males relative to noncircumcised males [6, 39, 40], while the baseline circumcision $f_{cm}$ was assumed to be 12.85%, based on Zambia’s Demographic and Health Survey (DHS) 2007 [41].

**References**

1. Alsallaq RA, Cash B, Weiss HA, Longini IM, Jr., Omer SB, Wawer MJ, et al. Quantitative assessment of the role of male circumcision in HIV epidemiology at the population level. Epidemics. 2009;1(3):139-52. Epub 2009/09/01. doi: 10.1016/j.epidem.2009.08.001. PubMed PMID: 21352761.

2. Garnett GP, Anderson RM. Balancing sexual partnerships in an age and activity stratified model of HIV transmission in heterosexual populations. IMA J Math Appl Med Biol. 1994;11(3):161-92. Epub 1994/01/01. PubMed PMID: 7822888.

3. Garnett GP, Anderson RM. Factors controlling the spread of HIV in heterosexual communities in developing countries: patterns of mixing between different age and sexual activity classes. Philos Trans R Soc Lond B Biol Sci. 1993;342(1300):137-59. Epub 1993/10/29. doi: 10.1098/rstb.1993.0143. PubMed PMID: 7904355.

4. Abu-Raddad LJ, Longini IM, Jr. No HIV stage is dominant in driving the HIV epidemic in sub-Saharan Africa. AIDS. 2008;22(9):1055-61. Epub 2008/06/04. doi: 10.1097/QAD.0b013e3282f8af84. PubMed PMID: 18520349.

5. Awad SF, Abu-Raddad LJ. Could there have been substantial declines in sexual risk behavior across sub-Saharan Africa in the mid-1990s? Epidemics. 2014;8:9-17.

6. Auvert B, Taljaard D, Lagarde E, Sobngwi-Tambekou J, Sitta R, Puren A. Randomized, controlled intervention trial of male circumcision for reduction of HIV infection risk: the ANRS 1265 Trial. PLoS Med. 2005;2(11):e298. PubMed PMID: 16231970.

7. Bailey R, editor Scaling up circumcision Programmes: The Road from Evidence to Practice. 4th IAS Conference on HIV Pathogenesis, Treatment & Prevention, July 22-25 2007, Sydney, Australia; 2007.

8. Gray RH, Li X, Kigozi G, Serwadda D, Nalugoda F, Watya S, et al. The impact of male circumcision on HIV incidence and cost per infection prevented: a stochastic simulation model from Rakai, Uganda. AIDS. 2007;21(7):845-50. PubMed PMID: 17415039.

9. Cuadros DF, Crowley PH, Augustine B, Stewart SL, Garcia-Ramos G. Effect of variable transmission rate on the dynamics of HIV in sub-Saharan Africa. BMC Infect Dis. 2011;11:216. Epub 2011/08/13. doi: 10.1186/1471-2334-11-216. PubMed PMID: 21834977; PubMed Central PMCID: PMC3175213.

10. Handcock MS, Jones JH. Likelihood-based inference for stochastic models of sexual network formation. Theor Popul Biol. 2004;65(4):413-22. Epub 2004/05/12. doi: 10.1016/j.tpb.2003.09.006. PubMed PMID: 15136015.

11. Hamilton DT, Handcock MS, Morris M. Degree distributions in sexual networks: a framework for evaluating evidence. Sex Transm Dis. 2008;35(1):30-40. Epub 2008/01/25. PubMed PMID: 18217224.

12. Bansal S, Grenfell BT, Meyers LA. When individual behaviour matters: homogeneous and network models in epidemiology. J R Soc Interface. 2007;4(16):879-91. Epub 2007/07/21. doi: 10.1098/rsif.2007.1100. PubMed PMID: 17640863; PubMed Central PMCID: PMC2394553.

13. Kretzschmar M, Morris M. Measures of concurrency in networks and the spread of infectious disease. Mathematical Biosciences. 1996;133(2):165-95. PubMed PMID: ISI:A1996UD81200003.

14. Morris M. Sexual networks and HIV. Aids. 1997;11:S209-S16. PubMed PMID: ISI:A1997YJ01200028.

15. Watts CH, May RM. The influence of concurrent partnerships on the dynamics of HIV/AIDS. Math Biosci. 1992;108(1):89-104. Epub 1992/02/01. PubMed PMID: 1551000.

16. Kretzschmar M, Morris M. Measures of concurrency in networks and the spread of infectious disease. Math Biosci. 1996;133(2):165-95. Epub 1996/04/15. PubMed PMID: 8718707.

17. Morris M. Sexual networks and HIV. AIDS. 1997;11 Suppl A:S209-16. Epub 1997/01/01. PubMed PMID: 9451987.

18. Rao A, Iqbal M, Price DE, Stephens JW. Necrotizing fasciitis and hypothyroidism. QJM : monthly journal of the Association of Physicians. 2007;100(8):533-4. Epub 2007/06/05. doi: 10.1093/qjmed/hcm048. PubMed PMID: 17545683.

19. May RM, Anderson RM. The Transmission Dynamics of Human Immunodeficiency Virus (Hiv). Philosophical Transactions of the Royal Society of London Series B-Biological Sciences. 1988;321(1207):565-607. PubMed PMID: ISI:A1988Q802000014.

20. Ferry B, Carael M, Buve A, Auvert B, Laourou M, Kanhonou L, et al. Comparison of key parameters of sexual behaviour in four African urban populations with different levels of HIV infection. AIDS. 2001;15 Suppl 4:S41-50. Epub 2001/11/01. PubMed PMID: 11686464.

21. Lagarde E, Auvert B, Carael M, Laourou M, Ferry B, Akam E, et al. Concurrent sexual partnerships and HIV prevalence in five urban communities of sub-Saharan Africa. Aids. 2001;15(7):877-84. PubMed PMID: 11399960.

22. Awad SF, Cuadros DF, Abu-Raddad LJ. Generic patterns of HIV infection distribution in human populations. Under preparation. 2012.

23. Liljeros F, Edling CR, Amaral LAN, Stanley HE, Åberg Y. The web of human sexual contacts. Promiscuous individuals are the vulnerable nodes to target in safe-sex campaigns.2001; 411.

24. Barrat A, Barthelemy M, Pastor-Satorras R, Vespignani A. The architecture of complex weighted networks. Proc Natl Acad Sci U S A. 2004;101(11):3747-52. Epub 2004/03/10. doi: 10.1073/pnas.0400087101. PubMed PMID: 15007165; PubMed Central PMCID: PMC374315.

25. Boccaletti S, Latora V, Moreno Y, Chavez M, Hwang DU. Complex networks: Structure and dynamics. Physics Reports. 2006;424(4–5):175-308. doi: 10.1016/j.physrep.2005.10.009.

26. Watts DJ, Strogatz SH. Collective dynamics of 'small-world' networks. Nature. 1998;393(6684):440-2. Epub 1998/06/12. doi: 10.1038/30918. PubMed PMID: 9623998.

27. Barabási AL. Linked: how everything is connected to everything else and what it means for business, science and everyday life: London: First Plume Printing; 2003.

28. UNAIDS/WHO. AIDS epidemic update 2010: UNAIDS fact sheet 2010. Available: <http://www.unaids.org/documents/20101123_FS_SSA_em_en.pdf>.

29. UNAIDS. UNAIDS Report on the Global AIDS Epidemic 2010 2010. Available from: <http://www.unaids.org/globalreport/Global_report.htm>.

30. Mahboob A, Haroon TS, Iqbal Z, Saleemi MA, Munir A. Prevalence of hepatitis B surface antigen carrier state in patients with lichen planus--report of 200 cases from Lahore, Pakistan. Journal of Ayub Medical College, Abbottabad : JAMC. 2007;19(4):68-70. Epub 2008/08/13. PubMed PMID: 18693602.

31. Velicia FJF. On the moments of a Wood Saxon beta distribution. Journal of Physics A: Mathematical and General. 1987.

32. Woods RD, Saxon DS. Diffuse Surface Optical Model for Nucleon-Nuclei Scattering. Physical Review. 1954;95(2):577-8. doi: Doi 10.1103/Physrev.95.577. PubMed PMID: WOS:A1954UB48500064.

33. Pinkerton SD. Probability of HIV transmission during acute infection in Rakai, Uganda. AIDS Behav. 2008;12(5):677-84. Epub 2007/12/08. doi: 10.1007/s10461-007-9329-1. PubMed PMID: 18064559.

34. Hollingsworth TD, Anderson RM, Fraser C. HIV-1 transmission, by stage of infection. J Infect Dis. 2008;198(5):687-93. Epub 2008/07/30. doi: 10.1086/590501. PubMed PMID: 18662132.

35. Wawer MJ, Gray RH, Sewankambo NK, Serwadda D, Li X, Laeyendecker O, et al. Rates of HIV-1 transmission per coital act, by stage of HIV-1 infection, in Rakai, Uganda. J Infect Dis. 2005;191(9):1403-9. Epub 2005/04/06. doi: 10.1086/429411. PubMed PMID: 15809897.

36. UNAIDS. UNAIDS Reference Group on Estimates, Modelling and Projections. 2007.

37. UNAIDS/WHO. *AIDS epidemic update 2007*.

38. UNAIDS. Epidemiological data, HIV estimates 1990-2013. 2013. Available: <http://www.unaids.org/en/dataanalysis/datatools/aidsinfo>.

39. Bailey RC, Moses S, Parker CB, Agot K, Maclean I, Krieger JN, et al. Male circumcision for HIV prevention in young men in Kisumu, Kenya: a randomised controlled trial. Lancet. 2007;369(9562):643-56. Epub 2007/02/27. doi: 10.1016/S0140-6736(07)60312-2. PubMed PMID: 17321310.

40. Gray RH, Kigozi G, Serwadda D, Makumbi F, Watya S, Nalugoda F, et al. Male circumcision for HIV prevention in men in Rakai, Uganda: a randomised trial. Lancet. 2007;369(9562):657-66. Epub 2007/02/27. doi: 10.1016/S0140-6736(07)60313-4. PubMed PMID: 17321311.

41. Zambia Demographic and Health Survey 2007. Available: <http://dhsprogram.com/pubs/pdf/FR211/FR211%5Brevised-05-12-2009%5D.pdf> [Internet]. CSO and Macro International Inc. 2009.
