# Supplementary material for: Investigating Voluntary Medical Male Circumcision Program Efficiency Gains through Subpopulation Prioritization: Insights from Application to Zambia
Source: PLoS One. 2015 Dec 30;10(12):e0145729. doi: 10.1371/journal.pone.0145729 (PMC4696770; doi:10.1371/journal.pone.0145729)
Supplement: S2 Text — (DOCX) [file pone.0145729.s015.docx]

**Text S2**

**Inclusion of Provinces for Geographic Prioritization**

We analyzed both 2011 HIV sentinel surveillance from antenatal clinics (HSS-ANC) data [1] and 2007 Demographic and Health Survey (DHS) data [2], for all provinces in Zambia, to determine the provinces with HIV prevalence higher than the national prevalence. In Figure S1 we compare HIV prevalence from the two datasets.

Both datasets agreed that the Copperbelt, Lusaka, Southern, and Western provinces had higher HIV prevalence than the national prevalence. However, according to DHS data, the Central province had the second-highest HIV prevalence, while according to HSS-ANC data it had the second-lowest HIV prevalence. Looking at the spatial distribution of high HIV prevalence clusters in Zambia published by Cuadros et al. [3], based on analyses of DHS data, the Central province had a high HIV prevalence cluster. Accordingly, the Central province was included in the geographic prioritization analysis, as the province with the second-highest HIV prevalence in Zambia.

HIV prevalence in the Southern province was slightly higher than the national prevalence, and the clustering analysis showed that it contained one cluster of low HIV prevalence and a very small cluster of high HIV prevalence. Taking this and the relatively large size of the population in this province into account (Figure S2), we did not include the Southern province as one of those to be prioritized in the geographical analysis.

Based on the above, we included Central, Copperbelt, Lusaka, and Western provinces in the geographic prioritization analysis to be the ones with HIV prevalence higher than the national prevalence (scenario 1); Central and Lusaka provinces to be the two provinces with the highest HIV prevalence (scenario 2); and Lusaka province to be the province with the highest HIV prevalence (scenario 3).

**References**

1. Vandament L. Zambia 2011 HIV sentinel surveillance data from antenatal clinics. Country-level Data, Lusaka, Zambia 2013.

2. Zambia Demographic and Health Survey 2007. Available: <http://dhsprogram.com/pubs/pdf/FR211/FR211%5Brevised-05-12-2009%5D.pdf> [Internet]. CSO and Macro International Inc. 2009.

3. Cuadros DF, Awad SF, Abu-Raddad LJ. Mapping HIV clustering: a strategy for identifying populations at high risk of HIV infection in sub-Saharan Africa. International journal of health geographics. 2013;12:28. Epub 2013/05/23. doi: 10.1186/1476-072X-12-28. PubMed PMID: 23692994; PubMed Central PMCID: PMC3669110.
